# A pragmatic trial of glucocorticoids for community acquired pneumonia

**DOI:** 10.1056/NEJMoa2507100

**Published:** 2025-10-29

**Authors:** Ruth Khadembu Lucinde, Henry Gathuri, Paul Mwaniki, Benedict Orindi, Edwin Onyango Otieno, Stella Mwakio, Lillian Mulemi, Lynda Isaaka, Jimmy Shangala, Metrine Saisi, Elizabeth Isinde, Irene Njeri Oginga, Alvin Waweru Wachira, Evans Manuthu, Hazel Kariuki, Patrick Asaava, Jared Nyikuli, Cyprian Wekesa, Amos Otedo, Hannah Bosire, Steve Biko Okoth, Winston Ongalo, David Mubashi Mukabi, Wilberforce Lusamba, Beatrice Muthui, Isaac Adembesa, Caroline Mithi, Mohammed Sood, Nadia Ahmed Aliyan, Bernard Gituma, Matiko Giabe Matiko, Charles Ayoro Omondi, Loice Achieng Ombajo, Nicholas Kirui, Lucy Ochola, Abdirahman I. Abdi, Eunice Wangeci Kagucia, Mike English, Mainga Hamaluba, Isabella Ochola-Oyier, Dorcas Kamuya, Philip Bejon, Edwine Barasa, Ambrose Agweyu, Samuel Akech, Anthony Oliwa Etyang

**Affiliations:** 1. Kenya Medical Research Institute-Wellcome Trust Research Programme, Kilifi, Kenya; 2. Kiambu Level Five Hospital, Kiambu, Kenya; 3. Machakos Level Five Hospital, Machakos, Kenya; 4. Kitale County Referral Hospital, Kitale, Kenya; 5. Naivasha Level Five Hospital, Naivasha, Kenya; 6. Bungoma County Referral Hospital, Bungoma, Kenya; 7. Kisumu County Hospital, Kisumu, Kenya; 8. Kakamega County General Hospital, Kakamega, Kenya; 9. Busia County Referral Hospital, Busia, Kenya; 10. Mama Lucy Kibaki Hospital, Nairobi, Kenya; 11. Kenyatta University Teaching and Referral Hospital, Nairobi, Kenya; 12. Coast General Teaching and Referral Hospital, Mombasa, Kenya; 13. Kilifi County Hospital, Kilifi, Kenya; 14. Mbagathi County Hospital, Nairobi, Kenya; 15. Kisii Teaching and Referral Hospital, Kisii, Kenya; 16. Jaramogi Oginga Odinga Teaching and Referral Hospital, Kisumu, Kenya; 17. Kenyatta National Hospital, Nairobi, Kenya; 18. The University of Nairobi, Nairobi, Kenya; 19. Moi Teaching and Referral Hospital, Eldoret, Kenya; 20. Institute of Primate Research, Nairobi, Kenya; 21. Centre for Tropical Medicine & Global Health, Nuffield Department of Medicine, University of Oxford, United Kingdom; 22. London School of Hygiene and Tropical Medicine, London, United Kingdom

## Abstract

**Background:**

Adjunctive glucocorticoids may reduce mortality among patients with severe community acquired pneumonia (CAP) in well-resourced settings. It is unclear if they are beneficial in low-resource settings with limited diagnostic and treatment facilities.

**Methods:**

In this randomized, controlled, open label pragmatic trial conducted across 18 public hospitals in Kenya, we assigned adult participants within 48 hours of admission to the general medical wards to receive either standard care for CAP or oral low-dose glucocorticoids for 10 days in addition to standard care. The primary outcome was mortality 30 days after enrollment.

**Results:**

We enrolled 2180 participants:1089 to the glucocorticoid arm and 1091 to the standard care arm. The median age was 53.0 years (IQR 38–72 years), and 1009 (46%) were women. Participant characteristics were evenly distributed by study arm. At day 30, 530 (24.3%) participants had died, 246 (22.6%) of 1089 in the glucocorticoid arm and 284 (26.0%) of 1091 in the standard care arm; Hazard Ratio, 0.84; 95% confidence interval (CI), 0.73 to 0.97, P=0.021. The incidences of adverse events and serious adverse events (SAEs) were similar in the intervention and standard care arms. Five participants (0.4%) experienced SAEs that were related to glucocorticoid administration.

**Conclusion:**

Adjunctive glucocorticoid therapy among patients with community-acquired pneumonia in a low-resource setting was associated with a reduction in mortality compared to standard care. (Funded by Wellcome Trust and others; PACTR number PACTR202111481740832, ISRCTN number ISRCTN36138594)

## Background

Community-acquired pneumonia (CAP) is a leading cause of morbidity and mortality globally^1^. Case fatality due to CAP in sub-Saharan Africa (sSA) is 3–5 times higher than that in high-income settings despite patients being significantly younger^1,2^.

Glucocorticoids have been proposed as adjunctive therapy for CAP due to their immunomodulatory effects^3^. Data from two recently published trials^4 5^ and systematic reviews of adjunctive hydrocortisone in CAP requiring intensive care unit (ICU) admission ^6–8^ , indicate reduced mortality among these patients. However, uncertainty remains as other studies^4,9 10^ showed no benefit. Some^3^ but not all^11^ guidelines have been updated to recommend glucocorticoids for selected groups with CAP, but it is unclear what the risks and benefits would be for patients in low-resource settings such as sSA. First, previous trials were conducted among significantly older patients than those in sSA, excluding patients with comorbidities that are common among CAP patients in sSA such as human immunodeficiency virus (HIV) infection and pulmonary tuberculosis. Second, delayed presentation to hospital, common in sSA, could compromise the effectiveness of adjunctive glucocorticoids for CAP patients, as evidence suggests that they should be given early in the course of the disease^4,5^. Third, limitations in diagnostic capacity in low-resource settings make it difficult to stratify patients by severity of their CAP, which appears to influence glucocorticoid efficacy. Finally, most of the evidence showing mortality reduction with use of adjunctive glucocorticoids is derived from studies conducted in critical care units, non-ICU based studies did not have mortality as a primary endpoint^12,13^. The limited availability of ICUs in sSA^14^ constrains the ability to identify and treat severely ill patients and manage any adverse events that might result from glucocorticoid administration.

We conducted a pragmatic randomized controlled trial to evaluate the effectiveness and safety of adjunctive low-dose glucocorticoids among adult patients hospitalized with CAP in Kenya.

## Methods

### Trial design and participants

The Steroids in Pneumonia (SONIA) trial^15^ was a randomized, controlled, pragmatic trial conducted in 18 first-level referral hospitals (Figure S1) that are part of a clinical information network in Kenya^16^. Access to critical care units was very limited to non-existent in these hospitals (Table S4), and patients were recruited from the general medical wards. Eligible patients were adults (aged 18 years or older) with a diagnosis of CAP who did not have a clear indication for glucocorticoids to be included as part of their treatment. CAP was defined as the presence of at least 2 of the following signs and symptoms for less than 14 days: cough, fever, dyspnea, hemoptysis, chest pain or crackles on chest examination. Participants were enrolled within the first 48 hours of admission to hospital. Patients were excluded if they had a contraindication to glucocorticoids, were pregnant or breastfeeding, had hospital acquired pneumonia or had a known or suspected condition requiring glucocorticoids e.g., asthma or COVID-19 (supplementary appendix page 2). Assessment of etiology of CAP through imaging or laboratory tests and standard severity scoring are frequently unavailable at the participating centers and were not a prerequisite for enrollment in this trial.

### Trial procedures and follow-up

Participants in this open label trial were randomly assigned in a 1:1 ratio to receive either standard care for CAP (control arm) or standard care for CAP plus adjunctive low-dose glucocorticoids (intervention arm). A randomization list was prepared centrally by an independent trial statistician prior to recruitment, who sealed randomization cards in opaque envelopes. Sites received batches of these sealed envelopes, which were securely stored, and opened sequentially only after confirming participant eligibility and enrolment. Standard care was determined by attending physicians and included a beta-lactam (such as benzyl penicillin or a cephalosporin) and a macrolide (typically erythromycin or azithromycin) per World Health Organization guidelines^17^. Those assigned to the intervention arm were further randomized to receive a single daily dose of one of five locally available glucocorticoids in bioequivalent doses for a total of 10 days, including after discharge, in addition to standard care. These were dexamethasone 6mg, hydrocortisone 160mg, methylprednisolone 30mg, prednisolone 50mg or prednisone 50mg (Table S5). The dose and duration of glucocorticoid therapy was informed by the RECOVERY trial^18^. Where oral administration of glucocorticoids was not possible at enrollment, intravenous formulations were administered until when it was clinically possible to revert to oral formulations (Table S11). Due to the high pill burden imposed by locally available formulations of hydrocortisone and prednisone (Table S5), these were discontinued from the second month of recruitment. There was no tapering of the glucocorticoids at the end of treatment^19^.

The trial team provided trial glucocorticoids free of charge but did not influence any other aspects of patient management. During hospitalization, participants were followed up in-person daily by the trial team. Follow-up after discharge from hospital was through phone calls made to the participants or their next of kin on days 14 and 30 post-enrolment. A local clinical officer (non-physician clinician^20^) was employed at each hospital to conduct trial roles including recruitment and participant follow-up. This clinician had no role in patient management, which was done by the local hospital medical teams led by a consultant physician.

### Outcomes

The primary outcome for the trial was all-cause mortality 30 days after enrollment. Secondary outcomes included mortality at days 7, 14 and 21, in-hospital and after discharge from hospital (up to 30 days post enrollment). Safety outcomes comprised adverse events and serious adverse events. Results of an additional pre-specified secondary outcome examining immune responses by study arm will be reported later.

### Statistical analysis

We estimated that enrollment of 2180 patients would provide 85% power to detect a 25% relative reduction in mortality at day 30, assuming 20% mortality in the control arm and 15% in the glucocorticoid arm while allowing for 5% loss to follow-up^1,2,21^.

The statistical analysis plan was approved by the trial data and safety monitoring board (DSMB) prior to locking the database and embarking on analyses.

The primary analysis was a comparison of 30-day mortality in the intervention and control arms in the intention-to-treat population (all randomized patients in their assigned group). A Cox regression model incorporating study site as a stratification variable was used to estimate the hazard ratio (HR) for mortality and its associated 95% confidence interval. The proportional hazards assumption was assessed by plotting Schoenfeld residuals (Figure S3) and confirmed to be valid. Survival curves for the two trial arms were compared using a stratified log-rank test. Pre-specified subgroup analyses (age, sex, glucocorticoid type, study region, oxygen saturation at admission) were performed by including interaction terms in the regression models. Secondary outcome analyses included comparison of mortality at days 7, 14 and 21 since randomization and in-hospital and post-discharge mortality by study arm.

Statistical analyses for secondary endpoints have not been adjusted for multiple testing and the widths of the confidence intervals should not replace hypothesis testing. Safety analysis was performed on participants who received at least one dose of study treatment. Frequencies of adverse events were presented according to severity and relationship to treatment for participants in the intervention arm.

Following extensive missing data exploration our analysis was assumed valid under missing at random mechanism (see supplementary appendix page 3) ^22^. We conducted sensitivity analyses on two post hoc defined populations as follows: (1) complete-case analysis (excluded patients with missing 30-day outcome data), and (2) a modified intention-to-treat population (excluded patients who had received glucocorticoids prior to randomization).

### Trial Oversight

We obtained ethical approval from the Kenyan Medical Research Institute Scientific and Ethics Review Unit (SERU 4319), the Kenya Pharmacy and Poisons Board (ECCT/21/11/02), and the University of Oxford’s Tropical Research Ethics Committee (OxTREC 4–22). Additional approvals were received from all 18 study sites. Written informed consent was obtained from participants and/or their legally acceptable representative. An independent trial steering committee and DSMB provided trial and safety oversight. The DSMB reviewed the results of an interim analysis (see supplementary appendix page 33) conducted after approximately half of the target number of primary events had occurred with stopping guidelines based on the Haybittle-Peto rules^23^, and recommended continuation of the trial. The first and the last authors had access to all study data and vouch for the accuracy and completeness of the data and the analyses, and for adherence of the trial to the protocol (available at nejm.org). The funder of the study had no role in study design, data collection, data analysis, data interpretation, or writing of the report.

## Results

### Participants

**Figure 1:**
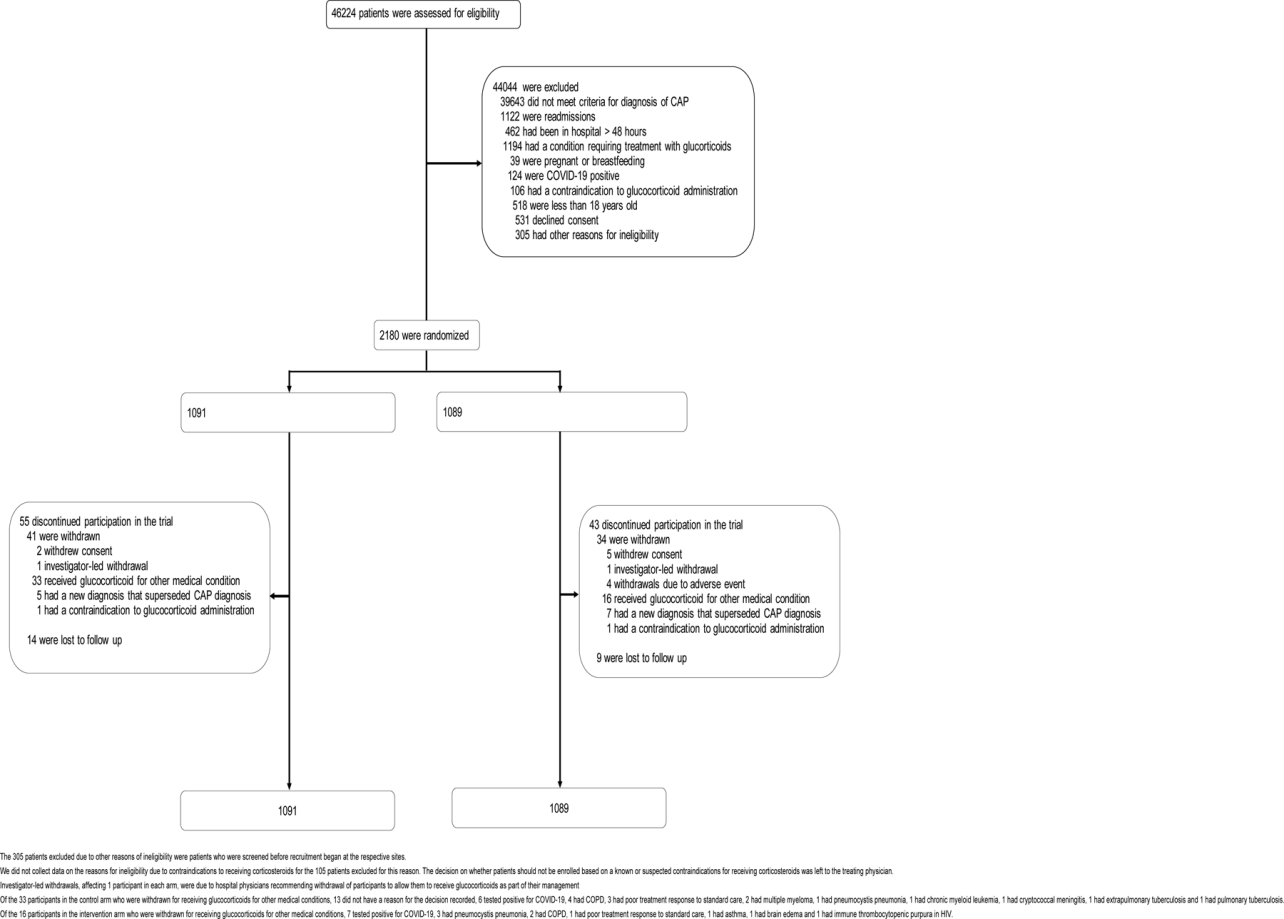
Screening, enrollment and follow up of trial participants The 305 patients excluded due to other reasons of ineligibility were patients who were screened before recruitment began at the respective sites. We did not collect data on the reasons for ineligibility due to contraindications to receiving corticosteroids for the 106 patients excluded for this reason. The decision on whether patients should not be enrolled based on a known or suspected contraindications for receiving corticosteroids was left to the treating physician. Investigator-led withdrawals, affecting 1 participant in each arm, were due to hospital physicians recommending withdrawal of participants to allow them to receive glucocorticoids as part of their management Of the 33 participants in the control arm who were withdrawn for receiving glucocorticoids for other medical conditions, 13 did not have a reason for the decision recorded, 6 tested positive for COVID-19, 4 had COPD, 3 had poor treatment response to standard care, 2 had multiple myeloma, 1 had pneumocystis pneumonia, 1 had chronic myeloid leukemia, 1 had cryptococcal meningitis, 1 had extrapulmonary tuberculosis and 1 had pulmonary tuberculosis. Of the 16 participants in the intervention arm who were withdrawn for receiving glucocorticoids for other medical conditions, 7 tested positive for COVID-19, 3 had pneumocystis pneumonia, 2 had COPD, 1 had poor treatment response to standard care, 1 had asthma, 1 had brain edema and 1 had immune thrombocytopenic purpura in HIV.

Recruitment took place from April 26, 2022 to June 30, 2024. A total of 46224 patients admitted to the adult medical wards of the participating hospitals were screened (Figure 1, Table S6). We enrolled 2180 participants, 1091 of whom were assigned to receive standard care, while 1089 were assigned to receive standard care plus glucocorticoids (Figure 1). Vital status (dead/alive) was known for 2107/2180 (96.7%) participants at day 14 and 2082/2180 (95.5%) participants at day 30. Of the 98 (4.5%) participants with missing day 30 data, 75 (3.4%) had been withdrawn from the trial and 23 (1.1%) were losses to follow-up (Figure 1). The median age at enrollment was 53.0 years (interquartile range 38–72 years), 1009 (46.3%) were women, and 808 (37.1%) had low oxygen saturation (SpO2 < 90%) at admission. The characteristics of participants at enrollment (Table 1), antibiotics received (Table S7) and adherence to prescribed medications (Table S12) were similar between the trial arms.

Participants included were considered representative of the general adult population with CAP at the trial locations (Table S9). The most common comorbidities at admission were HIV infection 344 (15.8%) and hypertension 300 (13.8%) (Table 1 and Table S10). Of the 1089 participants assigned to receive glucocorticoids, 352 (32.3%) were assigned to receive methyl prednisone, 343 (31.5%) to prednisolone, 371 (34.1%) to dexamethasone, and 23 (2.1%) to either hydrocortisone or prednisone (Table S11). The median duration of glucocorticoid treatment while in-patient was 4 (2–8) days (Table S7). Overall, 5 (0.2%) of all participants were transferred to an ICU during their hospital stay.

### Primary outcome

**Figure 2:**
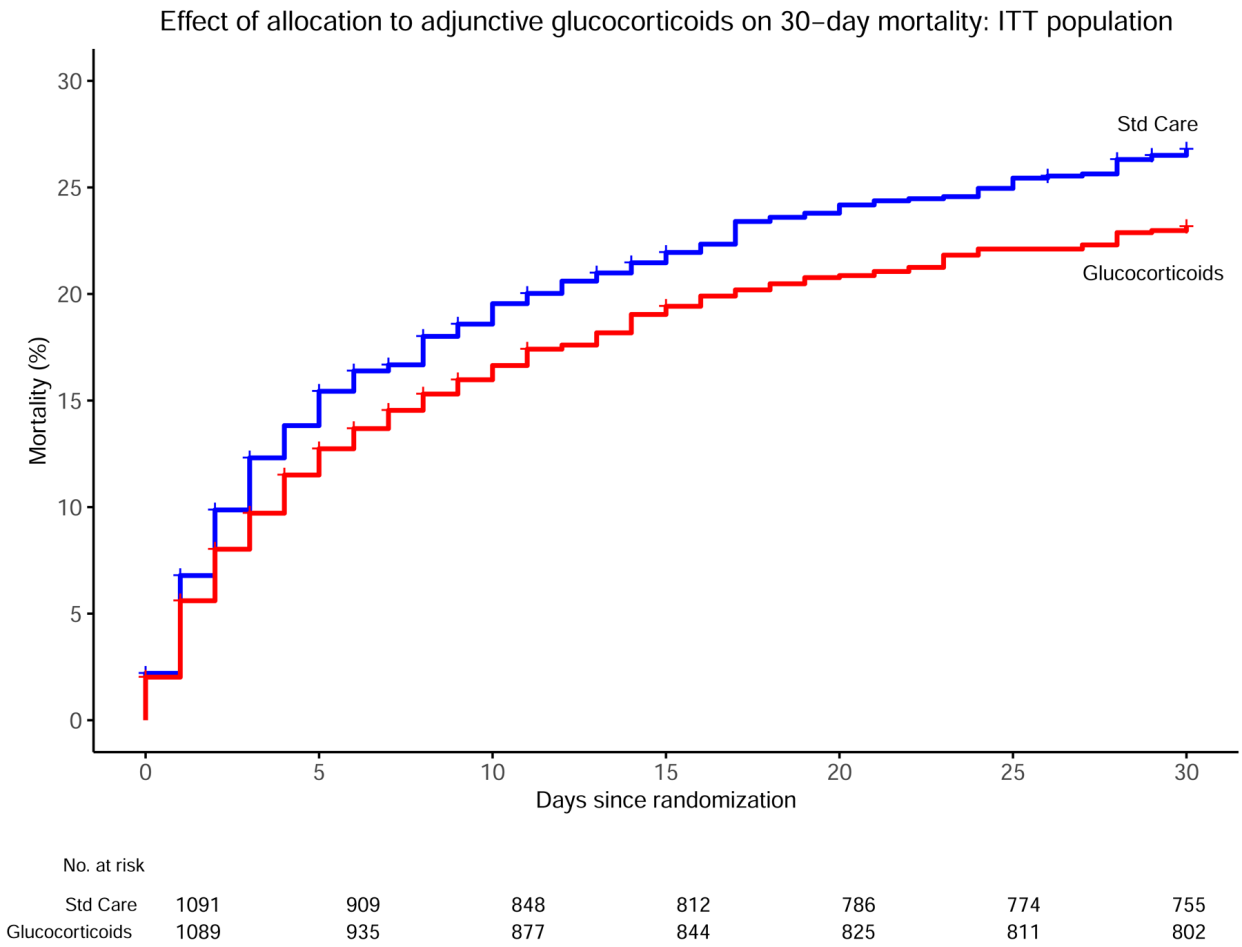
Cumulative all-cause mortality by study arm over 30 days in the intention to treat population Hazard Ratio derived from Cox regression comparing mortality between the two groups while accounting for study site was 0.84 (95% CI 0.72–0.97, P=0.021). The log-rank P-value for comparison of the two survival curves was 0.049

Of 2180 participants included in the ITT analyses, 530 (24.3%; 95% CI, 22.5–26.1%) died within the 30-day follow-up period. There were 246/1089 (22.6%; 95% CI 20.2–25.2) deaths in the intervention arm and 284/1091 (26.0%; 95% CI 23.5–28.7) deaths in the control arm. In the ITT analysis, participants receiving glucocorticoids had a lower 30-day mortality rate compared to those receiving standard care alone (hazard ratio 0.84 [95% CI, 0.73–0.97]; P=0.021; Figure 2).

### Secondary outcomes

**Figure 3:**
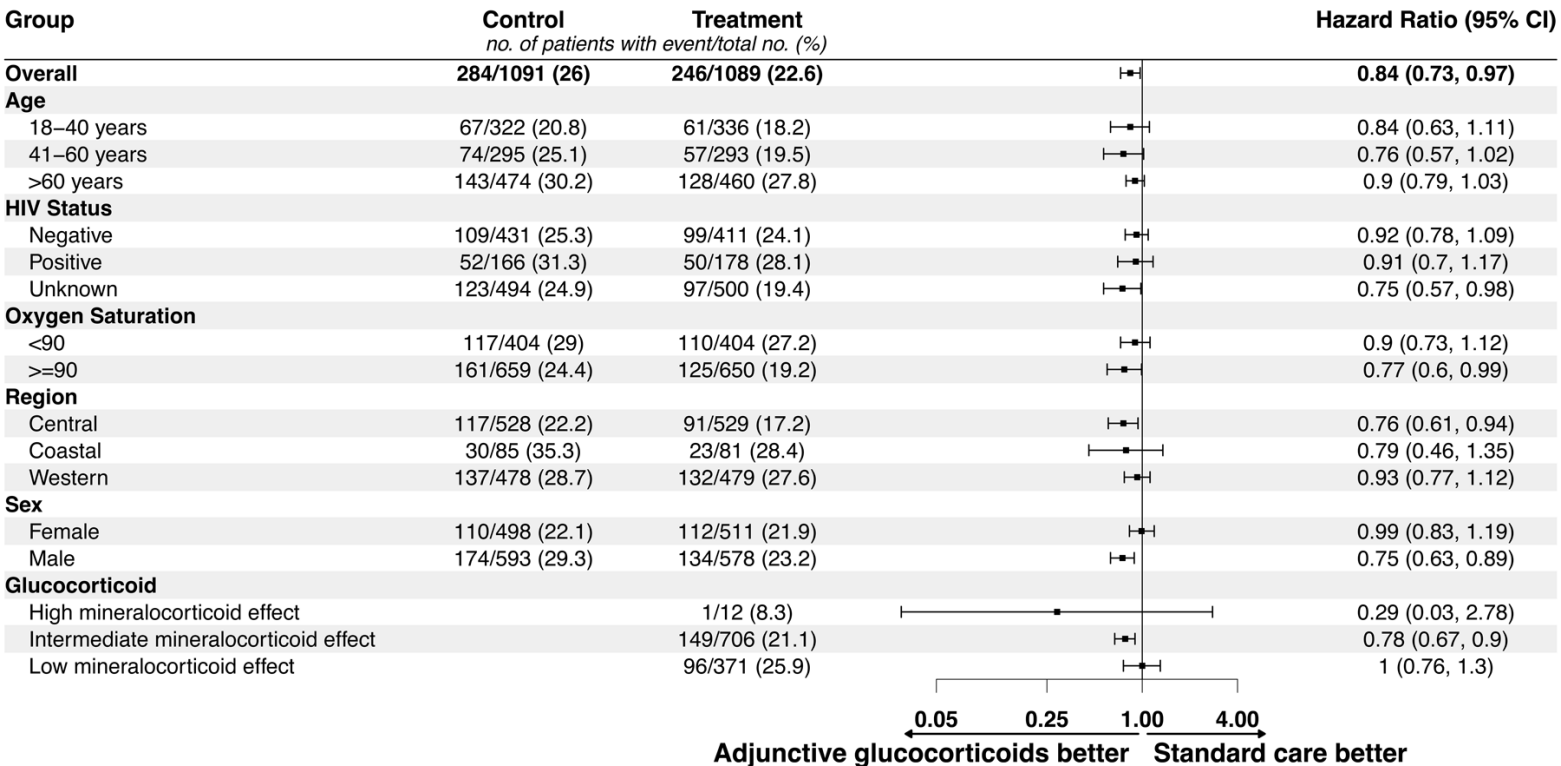
Forest plot summary of subgroup analyses Confidence intervals are not adjusted for multiplicity and should not be used in place of a hypothesis test Glucocorticoid categories by mineralocorticoid potency: High: Hydrocortisone; Intermediate: Methylprednisolone, prednisolone, prednisone; Low: Dexamethasone

Mortality at 7-,14- and 21- days after enrollment was consistent with the primary outcome (Table S13). There were 196/1089 (18%) in-hospital and 50/1089 (4.6%) out-of-hospital deaths respectively in the intervention arm and 223/1091 (20.4%) in-hospital and 61/1091(5.6%) out-of-hospital deaths respectively in the control arm (Table S14). Results stratified by the pre-specified subgroups are presented in Figure 3.

### Sensitivity analyses (Post hoc)

The Hazard Ratio in the complete case analysis (n=2082) was 0.84 (95% CI 0.73 – 0.96) while in the modified intention to treat population (n=2142) it was 0.83 (95% CI 0.72 – 0.97) (Table S15).

### Safety

A total of 385 adverse events in 338 participants were reported by day 30 after treatment. Of these, 18 (4.7%) were severe adverse events, 200 (51.9%) were mild and 167 (43.4%) were moderate events (Table 2). Of the 211 adverse events reported in the intervention arm, 62 (29.4%) were determined to be related to glucocorticoid administration. The most common adverse event diagnoses were pulmonary tuberculosis (35 events [20.1%]) and acute kidney injury (14 [8.0%]) in the control arm and pulmonary tuberculosis (34 [16.1%]) and hyperglycemia (35 [16.6%]) in the intervention arm (Table S17).

Ninety-six serious adverse events were reported throughout the study. Five of 1089 participants (<1%) in the intervention arm reported SAEs deemed possibly related to glucocorticoid administration (Table 2). The most common SAE in both groups was progression to severe CAP (13 events [17.1%]) (Table S18).

## Discussion

We report reduced all-cause mortality among patients with CAP randomized to receive adjunctive glucocorticoids within 48 hours of admission when implemented under pragmatic conditions in Kenya. While the effect size was lower than that in previous reports from France (HR 0.53)^5^, Egypt (HR 0.22)^24^ and from a meta-analysis of twelve trials (HR 0.62)^8^, our results are consistent in identifying a beneficial effect of glucocorticoids in the management of CAP.

Our trial is to the best of our knowledge, the largest and only one to date that has evaluated adjunctive glucocorticoids against a mortality endpoint among patients with CAP in a non-ICU setting. Previous trials of glucocorticoids in non-ICU settings conducted in Europe, reported reduced time to clinical stability^12^ and reduced length of stay and ICU admission rate^13^, but did not have mortality as a primary outcome. Of the eighteen studies informing the current Society of Critical Care Medicine guidelines that recommend the use of glucocorticoids in severe CAP^3^, only three were from Africa and they had a total of 194 patients, all of non-black ancestry ^24–26^. Compared to the CAPE-COD trial conducted in French ICUs^5^, the largest trial reporting a beneficial effect of hydrocortisone in reducing mortality among CAP patients, our participants were younger (median age 53 years versus 67 years), included more females (46.3% versus 30.6%), had more comorbidities causing immunosuppression at enrollment (16.3% versus 6.4%) and had higher mortality (24.3% versus 9.1%).

Although the primary result of this trial was partly accounted for by a higher mortality than what we had based our pre-trial power calculations on, we were not powered to assess differences in outcomes by glucocorticoid type or pneumonia severity. Therefore, potential differential effects by disease severity or glucocorticoid type in this setting remain uncertain. Additionally, the availability of cost-efficient and patient-friendly (pill burden) oral and intravenous formulations will need consideration if specific glucocorticoids are to be recommended in the treatment of CAP in our region^1^.

Glucocorticoids have been reported to be safe when used in the management of severe CAP, reversible hyperglycemia being the main side effect^7,8,27^. As reported elsewhere^5,28,29^, we report hyperglycemia as a common adverse event in this trial. The proportion of participants experiencing hyperglycemia as a SAE due to glucocorticoid administration was low (4%, Table S18), however, safety concerns remain and may need further investigation. We recommend accounting for the capacity to monitor blood sugar regularly if glucocorticoids are to be recommended for use in the management of CAP in sSA.

A major strength of our trial is the large sample size and large number of primary outcome events achieved with participants recruited from multiple sites representing diverse populations across the country. The pragmatic nature of this trial, designed to reflect real-world conditions in low-resource settings, means that the results are likely to be more relevant to sub-Saharan Africa settings. Based on our findings, adjunctive glucocorticoids could represent a low-cost intervention to reduce the high case fatality associated with CAP in sSA.

Our main limitation lies in the heterogenous patient population that we enrolled in the trial due to limited diagnostic and treatment capabilities. This constrained our ability to compare our trial participants with those in previous studies or identify which patients benefited from the intervention. It is possible that the results were affected by inclusion of patients for whom glucocorticoids have proven benefit e.g. pneumocystis pneumonia and septic shock.

However, the studies that showed benefit in these patients were conducted in markedly different settings and used different doses of glucocorticoids. Our broad eligibility criteria that ignored pneumonia severity and other baseline prognostic factors (e.g., functional status) may have biased our results toward the null given that glucocorticoids appear to have a larger effect among those with severe disease. We did not monitor co-interventions provided and the open-label nature of the trial could also have influenced the result, but this is mitigated by our use of a mortality endpoint.

Additionally, the time (in hours) to initiation of glucocorticoids could affect outcomes but was not recorded. Providing corticosteroids free of charge may have limited our ability to assess their effect under conditions where drug costs may influence treatment choices. Another limitation is that we primarily used oral formulations of glucocorticoids, limiting comparability with studies that used intravenous formulations with better treatment compliance.

## Conclusion

Adjunctive glucocorticoids among patients with community-acquired pneumonia in a low-resource setting was associated with a reduction in mortality.

Disclosure forms provided by the authors are available with the full text of this article at NEJM.org.

## Supplementary Material

Supplement

## Figures and Tables

**Table 1:** Characteristics of study participants at enrollment

Characteristic	Control arm (n=1091)	Intervention arm (n=1089)
Median Age (IQR), years	53.0 (38–72)	52.0 (38–72)
Sex, no. (%)		
Male	593 (54.4)	578 (53.1)
Female	498 (45.6)	511 (46.9)
Body mass index, kg/m^2^*	24.0±7.6	24.1±12.9
Chest X ray available^a^	425 (39.0)	423 (38.8)
O^2^ Saturation at admission, no. (%)		
SpO2 <90	404 (37.0)	404 (37.1)
SpO2 >=90	659 (60.4)	650 (59.7)
SpO2 Missing	28 (2.6)	35 (3.2)
Altered mental state	26 (2.4)	24 (2.2)
Systolic blood pressure		
< 90 mmHg	86 (7.9)	92 (8.4)
≥ 90 mmHg	1003 (91.9)	995 (91.4)
Missing	2 (0.2)	2 (0.2)
Respiratory rate		
< 30/minute	1016 (93.1)	996 (91.5)
≥ 30/minute	45 (4.1)	52 (4.8)
Missing	30 (2.7)	41 (3.8)
Temperature		
35.0°C - 39.9°C	1019 (93.4)	1008 (92.6)
< 35°C or ≥ 40°C	7 (0.6)	7 (0.6)
Missing	65 (6.0)	74 (6.8)
Pulse rate		
< 125/minute	978 (89.6)	930 (85.4)
≥ 125/minute	109 (10.0)	154 (14.1)
Missing	4 (0.4)	5 (0.5)
Random blood sugar		
< 14 mmol/L	975 (89.4)	993 (91.2)
≥ 14 mmol/L	51 (4.7)	31 (2.8)
Missing	65 (6.0)	65 (6.0)
HIV infection status, no. (%)		
Positive	166 (15.2)	178 (16.3)
Negative	431 (39.5)	411 (37.7)
Unknown	494 (45.3)	500 (45.9)
Chronic illness^†^, no. (%)	398 (36.0)	390 (35.8)
Hypertension	47 (4.3)	143 (13.1)
Diabetes mellitus	157 (14.4)	50 (4.6)
Congestive cardiac failure	70 (6.4)	17 (1.6)
Pulmonary Tuberculosis	21 (1.9)	21 (1.9)
Others	10 (0.9)	42 (3.9)

* Plus-minus values are means ±SD. SD denotes standard deviation. IQR denotes interquartile range

a Chest x-rays were not reviewed by radiologists; no formal clinical reports were available for all chest x-rays

† More detailed breakdown of chronic illnesses present in trial participants can be found in Table S6

**Table 2:** Summary of safety analysis

Safety parameter	Control arm (n=1091)	Intervention arm (n=1089)	Total (n=2180)
**Adverse events (AEs)**			
No. reported	174	211	385
Participants with an AE, no (%)^†^	154 (14.1)	184 (16.9)	338 (15.5)
Serious adverse events (SAEs)**			
No. reported	51	45	96
Participants with SAEs, no (%)^†^	48 (4.4)	44 (4.0)	92 (4.2)
AE severity, no./no. of reported events (%)**			
Mild	83/174 (47.7)	117/211 (55.5)	200/385 (51.9)
Moderate	82/174 (47.1)	85/211 (40.3)	167/385 (43.4)
Severe	9/174 (5.2)	9/211 (4.3)	18/385 (4.7)
**AE severity, no. of participants (%)** ^**‡**^			
Mild	79 (7.2)	104 (9.6)	183 (8.4)
Moderate	77 (7.1)	79 (7.3)	156 (7.2)
Severe	9 (0.8)	8 (0.7)	17 (0.8)
**Adverse event related to glucocorticoid treatment, no./ no. reported of events** ^**§**^			
Related	*	62/211 (29.4)	
Probably related	*	15/211 (7.1)	
Not related	*	134/211 (63.5)	

† Participants who had one or more adverse events or serious adverse events were counted only once.

‡ Participants were counted only once within each severity grade or relatedness category.

§ The relatedness of an adverse event to trial treatment was assessed by the site investigators.

* Relatedness not assessed for participants in the standard care arm as they did not receive any intervention.

** Adverse events were classified as mild, moderate or severe based on the intensity of the symptoms. Severe adverse events are to be differentiated from Serious Adverse Events which are defined by impact of patient health/level of functioning were defined as events where the outcomes included death, life-threatening events, hospitalization, disability or permanent damage, or required intervention to prevent permanent impairment.
