# Supplementary material for: A pragmatic trial of glucocorticoids for community acquired pneumonia
Source: N Engl J Med. Author manuscript; Available in PMC 2025 Nov 28. (PMC12659994; doi:10.1056/NEJMoa2507100)
Supplement: Supplement [file NIHMS2106627-supplement-Supplement.pdf]

Table of Contents

Summary of Eligibility Criteria 2

Supplementary methods: Missing data handling 3

Figure S1: Map of Kenya illustrating the distribution of trial sites 4

Figure S2: Summary of trial procedures. 6

Figure S3: Schoenfeld residuals plot for proportional hazards assumption. 8

Table S1. Baseline characteristics of participants with and without day 30 outcome data 9

Table S2. Characteristics associated with missing outcome data 11

Table S3. Characteristics of participants included and excluded from modified intention to treat (ITT) analysis 12

Table S4: Profile of inpatient wards of SONIA trial sites. 14

Table S5: Glucocorticoids administered to participants randomized to the intervention arm 15

Table S6. Distribution of participants by trial site. 16

Table S7: Characteristics and duration of prescribed treatments by trial arm 18

Table S8: Antibiotic use by trial region 20

Table S9: Summary of representativeness of trial sites 21

Table S10: Distribution of chronic comorbidities at enrollment by trial arm 22

Table S11: Summary of glucocorticoids received by participants randomized to the intervention arm. .. 23

Table S12: Summary of adherence to prescribed medications§ 24

Table S13: Cox regression analysis of mortality at 7-, 14- and 21- days post-enrolment 25

Table S14: In-hospital and out-of-hospital mortality by trial arm 25

Table S15: Results of complete case analysis and modified intention to treat (ITT) analysis of the primary outcome 26

Table S16: Number of deaths per trial site 27

Table S17: Summary of all adverse events reported in the trial 28

Table S18: Summary of all serious adverse events reported in the trial 31

Interim Analysis Report 32

Author contributions 39

References 40

Summary of Eligibility Criteria

Inclusion criteria

- Adults aged 18 years or over
- Admitted to hospital with a diagnosis of community-acquired pneumonia. Pneumonia diagnosis based on a clinical definition - the presence of at least 2 of the following signs and symptoms for less than 14 days: cough, fever, dyspnea, hemoptysis, chest pain or crackles on chest examination
- Admitted to hospital within the previous 48 hours
- Provides written informed consent or deferred written informed consent for a legally acceptable representative where patients are too ill to provide informed consent

Exclusion criteria

- Hospital acquired pneumonia defined as pneumonia in a patient who has been in hospital for >48 hours who did not have the symptoms at admission
- Patients who in the opinion of the attending clinician, require to be treated with glucocorticoids
- Known or suspected condition which in the opinion of attending clinician requires treatment with glucocorticoids, including but not limited to chronic obstructive pulmonary disease, asthma, adrenal insufficiency, Pneumocystis Jirovecii pneumonia (PCP)
- If the treating clinician strongly suspects COVID-19 and wants to provide glucocorticoids to the patient because of this suspicion or, a diagnosis of COVID-19 confirmed via polymerase chain reaction (PCR) test of nasopharyngeal or oropharyngeal (NP/OP) swabs or, antigen rapid diagnostic tests (RDTs) for SARS-CoV-2
- Pregnancy or breast feeding
- Any contraindication to glucocorticoid administration

Supplementary methods: Missing data handling

We explored how much data was missing and in what patterns. We investigated whether the missing data values were missing completely at random (MCAR); that is, whether missingness was unrelated to the trial variables using the approach proposed by Little et al.(1). Logistic regression analysis was used to identify variables associated with dropout. The outcome variable in the logistic regression model was dropout taking on the value 0 if the participant did not miss the visit and 1 if the visit was missed. The covariates were treatment arm and the baseline characteristics listed in Table 1.

We found that generally our trial had a low attrition rate, with 3.3% (73/2180) participants missing day 14 visit, and 4.5% (98/2180) day 30 visit for unknown reasons. The missing data values were similarly distributed between the two treatment arms, and across the participant baseline characteristics (Tables S2). Little's MCAR test indicated that data were not MCAR (Chi square P<0.001). Logistic regression analysis results indicated dropout was association with neither trial arm nor baseline characteristics except body mass index (P=0.011).

These observations suggest the missing data mechanism missing at random (MAR; that is, recorded characteristics can account for differences in the distribution of missing variables for observed and missing cases) or missing not at random (MNAR; that is, recorded characteristics do not account for differences in the distribution of the missing variables for observed and missing cases), but it is not possible to evaluate whether the missing data are MAR or MNAR (2). Together, the non-differential rates of dropout across the treatment arms, the reasonably low levels of dropout observed on the primary outcome (<5%), and our analytical method that used all available data would lead to little bias to affect our findings and/or conclusions (3).

Figure S1: Map of Kenya illustrating the distribution of trial sites

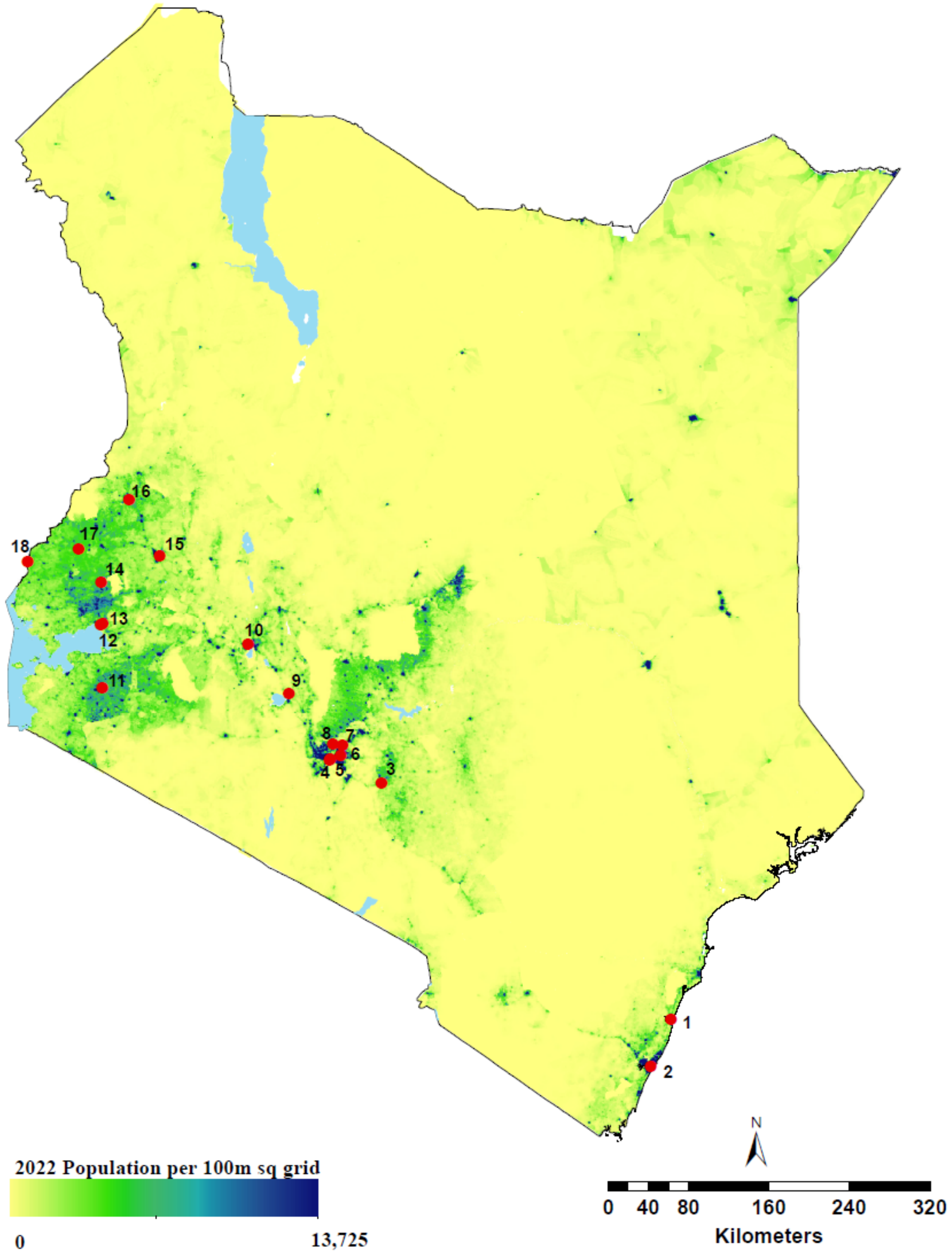

SONIA trial sites were distributed across densely populated areas of the country and are part of a clinical information network (16). Trial site names and region have been summarized in the table below:

Number on map	Region	Trial site
1	Coastal Kenya	Kilifi County Hospital
2	Coastal Kenya	Coast General Teaching and Referral Hospital
3	Central Kenya	Machakos County Referral Hospital
4	Central Kenya	Kenyatta National Hospital
5	Central Kenya	Mbagathi sub-County Hospital
6	Central Kenya	Mama Lucy Kibaki Hospital
7	Central Kenya	Kenyatta University Teaching Research and Referral Hospital
8	Central Kenya	Kiambu County Referral Hospital
9	Central Kenya	Naivasha County Hospital
10	Central Kenya	Nakuru County Referral Hospital
11	Western Kenya	Kisii County Referral Hospital
12	Western Kenya	Kisumu County Hospital
13	Western Kenya	Jaramogi Oginga Odinga Teaching and Referral Hospital
14	Western Kenya	Kakamega County Teaching and Referral Hospital
15	Western Kenya	Moi Teaching and Referral Hospital
16	Western Kenya	Kitale County Hospital
17	Western Kenya	Bungoma County Hospital
18	Western Kenya	Busia County Hospital

Figure S2: Summary of trial procedures.

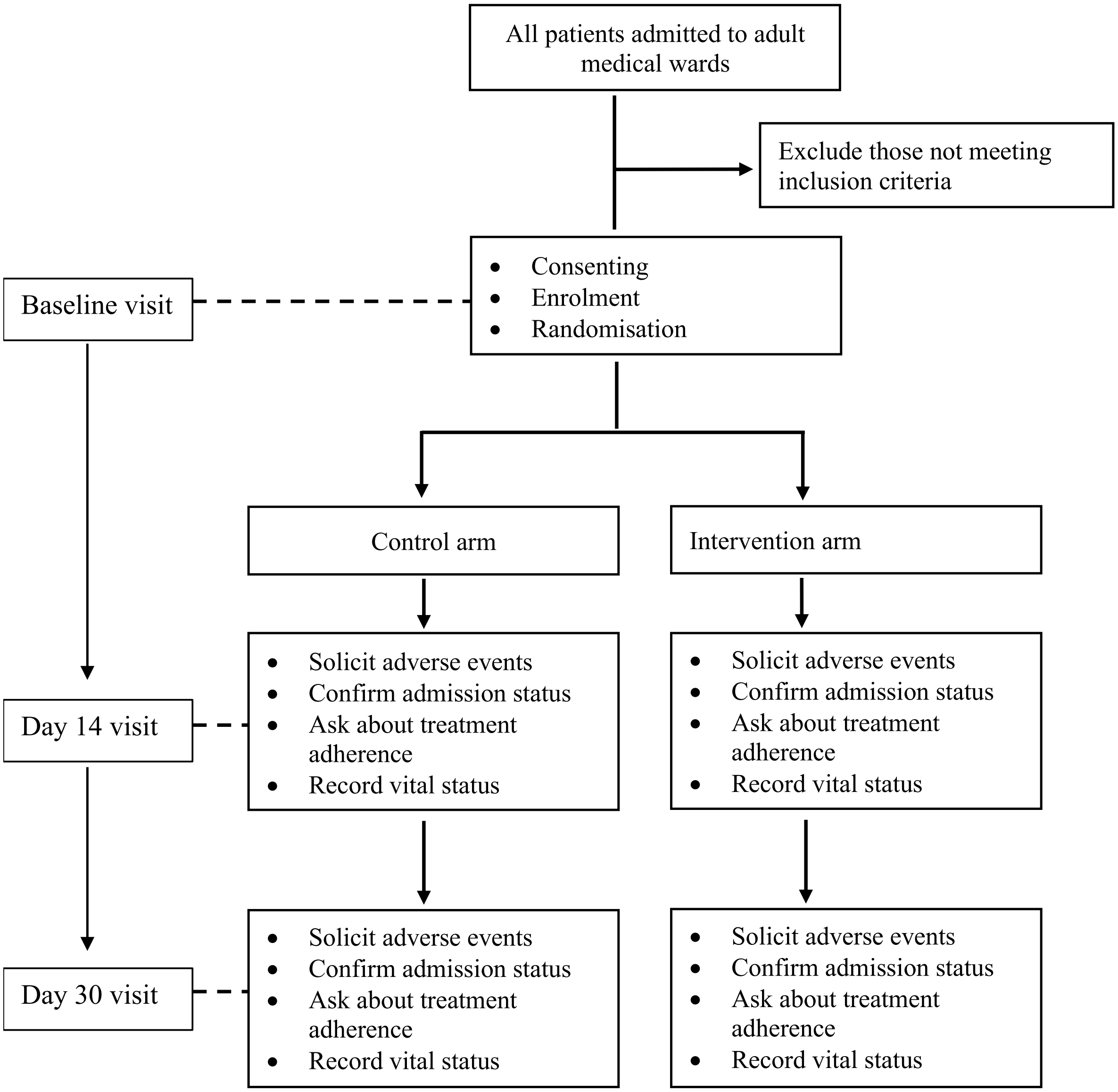

Summary of procedures in Figure S2:

- A randomization list was prepared centrally by an independent trial statistician prior to recruitment. Allocation was concealed using individually sealed, opaque envelopes, each containing a randomization card corresponding to the pre-generated list. Each study site received a batch of these sealed envelopes, which were securely stored in access-controlled cabinets. Trial clinicians were instructed to open the next sequentially numbered envelope only after confirming participant eligibility and enrolment. This ensured that allocation concealment was maintained at the point of assignment.

- To monitor adherence to the randomization sequence, clinicians entered each participant's randomization code into the electronic data capture system at the time of enrolment. These entries were regularly reviewed by the trial data manager to confirm that allocations followed the expected sequence. Investigators and clinical teams had no access to the master randomization list and were unable to anticipate a participant's allocation prior to opening the envelope
- All trial participants were followed up daily while in-patient and via phone call on the 14th and 30th day post enrolment if discharged.
- Treatment adherence to standard care and glucocorticoids was monitored daily by the trial team while in-patient and via phone call at the Day-14 and Day-30 visits.

Figure S3: Schoenfeld residuals plot for proportional hazards assumption.

The figure below illustrates that there was no evidence of violation of the proportional hazards assumption in assessing the primary outcome

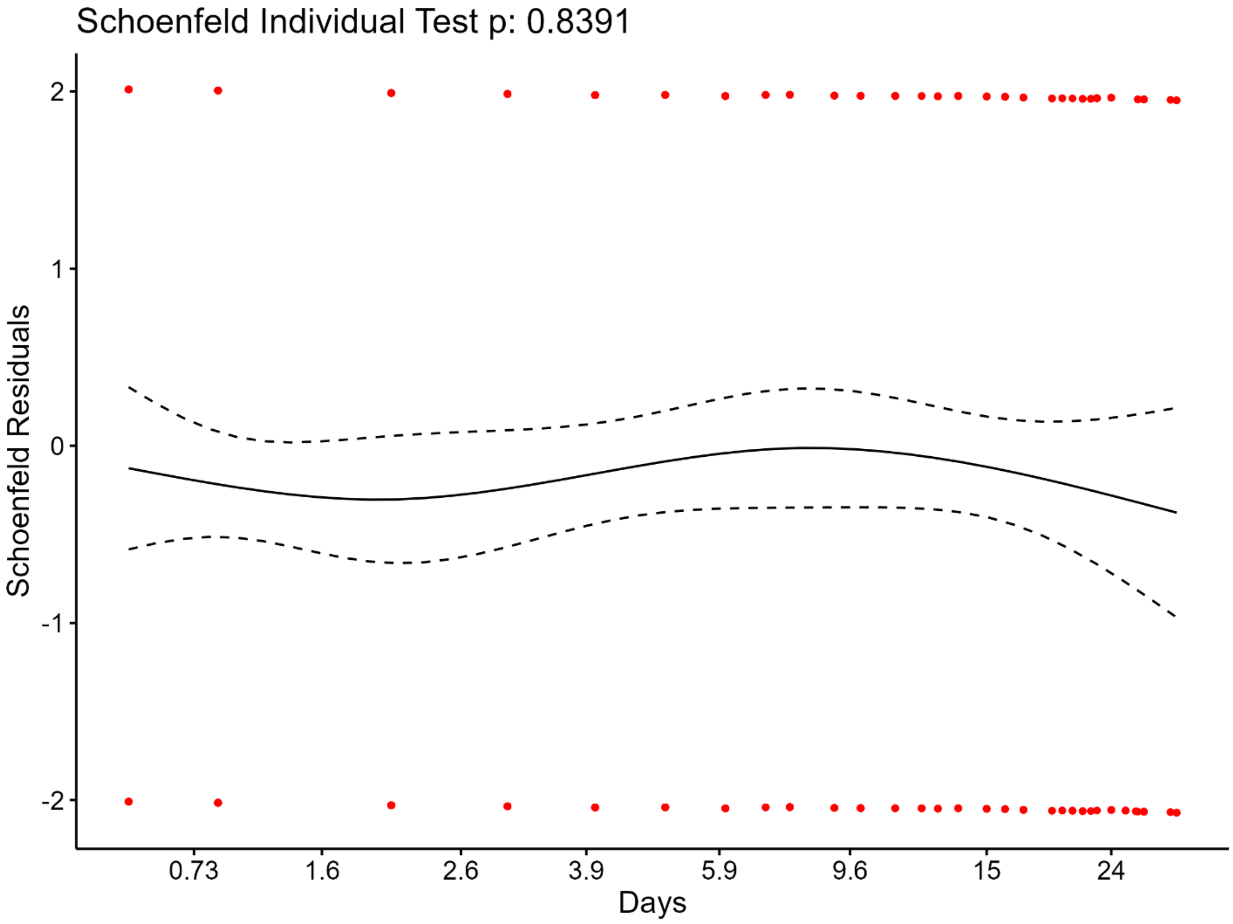

The proportional hazards assumption was met for the Cox regression model assessing the primary outcome, as indicated by the non-significant test of Schoenfeld residuals (P = 0.8391) and the absence of a systematic trend in the residuals over time.

Table S1. Baseline characteristics of participants with and without day 30 outcome data

Characteristic	Missing day 30 data (n=98)	Complete data (n=2082)
Trial arm, no. (%)		
Standard care	55/1091 (5.0)	1036/1091 (95.0)
Glucocorticoids	43/1089 (3.9)	1046/1089 (96.0)
Median Age (IQR), years	52.0 (36–71)	53.0 (38–72)
Sex, no. (%)		
Male	59 (60.2)	1112 (53.4)
Female	39 (39.8)	970 (46.6)
Body mass index, kg/m^2	21.9±4.5	24.15±10.5
Chest X ray available	43 (43.9)	805 (38.7)
O^2 Saturation at admission, no. (%)		
SpO2 <90	33 (33.7)	775 (37.2)
SpO2 >=90	61 (62.2)	1248 (59.9)
SpO2 Missing	4 (4.1)	59 (2.8)
Altered mental state	7 (7.1)	43 (2.1)
Systolic blood pressure		
< 90 mmHg	6.0 (6.1)	172 (8.3)
≥ 90 mmHg	91.0 (92.9)	1907 (91.6)
Missing	1.0 (1.0)	3 (0.1)
Respiratory rate		
< 30/minute	82 (83.7)	1930 (92.7)
≥ 30/minute	10 (10.2)	87 (4.2)
Missing	6 (6.1)	65 (3.1)
Temperature		
35.0ºC - 39.9ºC	88.0 (89.8)	1939 (93.1)
< 35ºC or ≥ 40ºC	-	14 (0.7)
Missing	10.0 (10.2)	129 (6.2)
Pulse rate		

< 125/minute	86.0 (87.8)	1822 (87.5)
≥ 125/minute	11.0 (11.2)	252 (12.1)
Missing	1.0 (1.0)	8 (0.4)
Random blood sugar		
< 14 mmol/L	85 (86.7)	1883 (90.4)
≥ 14 mmol/L	4 (4.1)	78 (3.7)
Missing	9 (9.2)	121 (5.8)
HIV infection status, no. (%)		
Positive	23 (23.5)	321 (15.4)
Negative	44 (44.9)	798 (38.3)
Unknown	31 (31.6)	963 (46.3)
Chronic illness[†], no. (%)	40 (40.8)	748 (35.9)

Table S2. Characteristics associated with missing outcome data

Characteristic	Odds ratio	95% Confidence Interval	p-value
Age	1.00	0.99, 1.02	0.5
Gender			
Female	—	—	
Male	1.32	0.82, 2.16	0.3
Trial arm			
Standard Care	—	—	
Standard Care plus Glucocorticoids	0.77	0.48, 1.22	0.3
Oxygen saturation at admission			
<90%	—	—	
>=90%	0.88	0.55, 1.45	0.6
Missing	1.91	0.29, 7.05	0.4
HIV status			
Negative	—	—	
Positive	0.98	0.43, 2.26	>0.9
Unknown	0.44	0.25, 0.75	0.003
Body Mass Index	0.93	0.88, 0.98	0.011
Chronic_illness			
No	—	—	
Yes	0.92	0.47, 1.68	0.8
Little's MCAR Test Results			
statistic	df	p.value	missing.patterns
49.73595	6	5.310308e-09	2

Table S3. Characteristics of participants included and excluded from modified intention to treat (ITT) analysis

Characteristic[±]	Excluded in modified ITT analysis	Included in modified ITT analysis
Trial arm, no. (%)		
Control arm	19/1091 (1.7)	1072/1091 (98.3)
Intervention arm	19/1089 (1.7)	1070/1089 (98.3)
Median Age (IQR), years	67.5 (49-75)	53.0 (38–72)
Sex, no. (%)		
Male	19(50.0%)	1152 (53.8%)
Female	19(50.0%)	990 (46.2%)
Body mass index, kg/m^2	23.8±2.9	24.1±10.4
Chest X ray available	12(31.6%)	836 (39.0%)
O^2 Saturation at admission, no. (%)		
SpO2 <90	13.0 (34.2%)	795 (37.1%)
SpO2 >=90	25.0 (65.8%)	1284 (59.9%)
SpO2 Missing	-	63 (2.9%)
Altered mental state	-	50 (2.3%)
Systolic blood pressure		
< 90 mmHg	1.0 (2.6%)	177 (8.3%)
≥ 90 mmHg	37.0 (97.4%)	1961 (91.5%)
Missing	-	4 (0.2%)
Respiratory rate		
< 30/minute	35.0 (92.1%)	1977 (92.3%)
≥ 30/minute	3.0 (7.9%)	94 (4.4%)
Missing	-	71 (3.3%)
Temperature		
35.0ºC - 39.9ºC	38.0 (100.0%)	1989 (92.9%)
< 35ºC or ≥ 40ºC	-	14 (0.7%)

Missing	-	139 (6.5%)
Pulse rate		
< 125/minute	29.0 (76.3%)	1879 (87.7%)
≥ 125/minute	9.0 (23.7%)	254 (11.9%)
Missing	-	9 (0.4%)
Random blood sugar		
< 14 mmol/L	38.0 (100.0%)	1930 (90.1%)
≥ 14 mmol/L	-	82 (3.8%)
Missing	-	130 (6.1%)
HIV infection status, no. (%)		
Positive	5 (13.2%)	339 (15.8%)
Negative	23 (60.5%)	819 (38.2%)
Unknown	10 (26.3%)	984 (45.9%)
Chronic illness[†], no. (%)	14 (36.8%)	774(36.1%)

±Patients that were excluded from the modified intention to treat analysis are those that were found (after enrolment) to have received glucocorticoids prior to randomization and were therefore ineligible to have entered the trial. In all instances the glucocorticoids had been administered in the emergency department prior to admission to the medical wards

Table S4: Profile of inpatient wards of SONIA trial sites.

Trial Site	[a] Medical ward capacity	Presence of critical care units	[a, b]Critical care unit capacity
1. Kilifi County Hospital	64	Yes; ICU	28
2. Coast General Teaching and Referral Hospital	71	Yes; ICU	15
3. Machakos County Referral Hospital	60	Yes; ICU	5
4. Kenyatta National Hospital [c]	727	Yes; both	82
5. Mbagathi sub-County Hospital	60	No	0
6. Mama Lucy Kibaki Hospital	68	Yes; ICU	2
7. Kenyatta University T.R.R. Hospital	80	Yes; both	45
8. Kiambu County Referral Hospital	72	Yes; ICU	3
9. Naivasha County Hospital	78	Yes; ICU	2
10. Nakuru County Referral Hospital	144	Yes; ICU	6
11. Kisii County Referral Hospital	120	Yes; ICU	9
12 Kisumu County Hospital	47	No	0
13. Jaramogi O.O. T. R. Hospital	120	Yes; ICU	13
14. Kakamega C. Teaching and Referral Hospital	43	Yes; ICU	6
15. Moi Teaching and Referral Hospital [c]	196	Yes; ICU	62
16. Kitale County Hospital	66	Yes; ICU	4
17. Bungoma County Hospital	80	Yes; ICU	6
18. Busia County Hospital	66	No	0
All sites (total)	2162		288

[a] This refers to the number of beds available. A hospital bed is defined as one that is staffed and equipped for continuous inpatient care. Approximately 58% of hospital beds in Kenya have an oxygen supply (5).
[b] Critical care units refer to intensive care units (ICUs) or high dependency units (HDUs). An ICU bed refers to a ventilator-supported bed in a specialized hospital unit for critically ill patients with life-threatening conditions(5). Beds in these units are shared across all hospital departments (surgical, medical, obstetrics and paediatric) and are largely inaccessible to medical department patients. Hence, only 5/2180 (0.2%) of trial participants were transferred to the ICU or HDU during hospitalization
[c] No participants were recruited from these sites. See Table S6 below

Table S5: Glucocorticoids administered to participants randomized to the intervention arm

Glucocorticoid	Mineralocorticoid effect	Equivalent potency	Trial dose	[a] Pill count
Dexamethasone	0	0.75	6mg	1.5 tabs
Hydrocortisone	1	20	160mg	16 tabs
Methylprednisolone	0.5	4	30mg	7.5 tabs
Prednisolone	0.8	5	50mg	2.5 tabs
Prednisone	0.8	5	50mg	10 tabs
Betamethasone	0	Not provided	5 mg	[b] Not applicable

Participants in the intervention arm were randomly assigned to receive any of the glucocorticoids except betamethasone We used doses that were bioequivalent to 6 mg Dexamethasone (6, 7).
[a] Pill count of bioequivalent dose based on locally available oral glucocorticoid formulations
[b] There are no locally available oral formulations of betamethasone in Kenya

Table S6. Distribution of participants by trial site.

[a]Trial site	Patients Screened	Patients with CAP n, [b](%)	Eligible n, [c](%)	Enrolled n, [d](%)	Missing day-30 outcome data n, (%)
1. Kilifi	3125	127 (4.1%)	59 (1.9%)	20 (33.9%)	3 (15.0%)
2. CPGH	3713	381 (10.3%)	198 (5.3%	146 (73.7%)	13 (8.9%)
3. Machakos	3435	254 (7.4%)	173 (5.0%)	157 (90.8%)	8 (5.1%)
4. Kenyatta	189	41 (21.7%)	7 (3.7%)	0 (0.0%)	0 (0%)
5. Mbagathi	1256	330 (26.3%)	99 (7.9%)	56 (56.6%)	1 (1.8%)
6. MLKH	1814	512 (28.2%)	165 (9.1%)	85 (51.5%)	6 (7.1%)
7. KUTRRH	1807	280 (15.5%)	83 (4.6%)	42 (50.6%)	8 (19.0%)
8. Kiambu	2801	485 (17.3%)	156 (5.6%)	120 (76.9%)	3 (2.5%)
9. Naivasha	4076	754 (18.5%)	517 (12.7%)	471 (91.1%)	7 (1.5%)
10. Nakuru	4209	617 (14.7%)	210 (5.0%)	126 (60.0%)	6 (4.8%)
11. Kisii	3822	163 (4.3%)	71 (1.9%)	67 (94.4%)	1 (1.5%)
12. Kisumu	1583	369 (23.3%)	175 (11.1%)	140 (80.0%)	6 (4.3%)
13. JOOTRH	1514	98 (6.5%)	50 (3.3%)	43 (86.0%)	4 (9.3%)
14. Kakamega	1693	324 (19.1%)	156 (9.2%)	118 (75.6%)	8 (6.8%)
15. Moi	1079	103 (9.5%)	1 (0.1%)	0 (0.0%)	0(0%)
16. Kitale	3418	655 (19.2%)	184 (5.4%)	85 (46.2%)	6 (7.1%)
17. Bungoma	3969	660 (16.6%)	468 (11.8%)	448 (95.7%)	13 (2.9%)
18. Busia	2721	428 (15.7%)	97 (3.6%)	56 (57.7%)	5 (8.9%)
**Total**	**46224**	**6581 (14.2%)**	**2869 (6.2%)**	**2180 (76.0%)**	**98 (4.5%)**

[a] Trial site complete names:

1- Kilifi County Hospital, 2- Coast General Teaching and Referral Hospital; 3- Machakos County Referral Hospital; 4- Kenyatta National Hospital; 5- Mbagathi sub-County Hospital; 6- Mama Lucy Kibaki Hospital; 7- Kenyatta University Teaching Research and Referral Hospital; 8- Kiambu County Referral Hospital; 9- Naivasha County Hospital; 10- Nakuru County Referral Hospital; 11- Kisii County Referral Hospital; 12- Kisumu County Hospital; 13- Jaramogi Oginga Odinga Teaching and Referral Hospital; 14- Kakamega County Teaching and Referral Hospital; 15- Moi Teaching and Referral Hospital; 16- Kitale County Hospital; 17- Bungoma County Hospital; 18- Busia County Hospital

[b] n refers to the number of screened patients who had a diagnosis of community acquired pneumonia (CAP); the % refers to the proportion of screened patients who had a diagnosis of CAP

[c] n refers to the number of screened patients who were eligible for recruitment; the % refers to the proportion of eligible patients from those who were screened

[d] n refers to the number of eligible patients recruited into the trial; the % refers to the proportion of recruited participants from those who were eligible

Table S7: Characteristics and duration of prescribed treatments by trial arm

Treatment/procedures	Control arm n (%)	Intervention arm n (%)	Total n (%)
**Antibiotic combinations received as part of standard care**			
Beta lactam + Macrolide	808 (74.1%)	826 (75.8%)	1634 (75.0%)
Beta lactam	125 (11.5%)	120 (11.0%)	245 (11.2%)
Bata lactam + Tetracycline	68 (6.2%)	64 (5.9%)	132 (6.1%)
Beta lactam + Beta lactam[a]	26 (2.4%)	16 (1.5%)	42 (1.9%)
Beta lactam + Nitroimidazole	17 (1.6%)	23 (2.1%)	40 (1.8%)
Macrolide	13 (1.2%)	7 (0.6%)	20 (0.9%)
Beta lactam + Sulfonamide	8 (0.7%)	8 (0.7%)	16 (0.7%)
Beta lactam + Azole	7 (0.6%)	6 (0.6%)	13 (0.6%)
Beta lactam + Aminoglycoside	3 (0.3%)	1 (0.1%)	4 (0.2%)
Azole	1 (0.1%)	1 (0.1%)	2 (0.1%)
Beta lactam + Nitrofurantoin	1 (0.1%)	0 (0.0%)	1(0.05%)
Beta lactam + Lincosamide	0 (0.0%)	1 (0.1%)	1(0.05%)
Beta lactam + Oxazolidinone	0 (0.0%)	1 (0.1%)	1(0.05%)
Macrolide + Azole	1 (0.1%)	0 (0.0%)	1(0.05%)
Macrolide + Oxazolidinone	0 (0.0%)	1 (0.1%)	1(0.05%)
Macrolide + Quinolone	1 (0.1%)	0 (0.0%)	1(0.05%)
Nitroimidazole	0 (0.0%)	1 (0.1%)	1(0.05%)
Quinolone + Nitroimidazole	0 (0.0%)	1 (0.1%)	1(0.05%)
Quinolone + Sulfonamide	0 (0.0%)	1 (0.1%)	1(0.05%)
Antibiotic data missing	12 (1.1%)	11 (1.0%)	23 (1.1%)
**Other treatments/procedures received as part of standard care**			
Glucocorticoids[±]	33 (3.0%)	16 (1.5%)	49 (2.2%)
Transferred to ICU	3 (0.3%)	2(0.2%)	5 (0.2%)

Duration (median, [IQR])			
Duration to receipt of trial glucocorticoids	N/A	[b]1 (1-1) day	[b]1 (1-1) day
Duration of hospitalization	5 (2-10) days	5 (2-9) days	5 (2-9) days
Duration of glucocorticoid administration while in-patient[c]	N/A	4 (2-8) days	4 (2-8) days

Decisions on the choice of antibiotics and other treatments provided to the patients were made by the hospital teams led by a consultant physician

[±]Refers to glucocorticoids that were prescribed by the clinical teams in the trial sites due to a clear indication for them arising after enrolment into the trial. These patients were withdrawn from the trial. Of the 33 participants in the control arm who were withdrawn for receiving glucocorticoids for other medical conditions, 13 did not have a reason for the decision recorded, 6 tested positive for COVID-19, 4 had COPD, 3 had poor treatment response to standard care, 2 had multiple myeloma, 1 had pneumocystis pneumonia, 1 had chronic myeloid leukemia, 1 had cryptococcal meningitis, 1 had extrapulmonary tuberculosis and 1 had pulmonary tuberculosis. Of the 16 participants in the intervention arm who were withdrawn for receiving glucocorticoids for other medical conditions, 7 tested positive for COVID-19, 3 had pneumocystis pneumonia, 2 had COPD, 1 had poor treatment response to standard care, 1 had asthma, 1 had brain edema and 1 had immune thrombocytopenic purpura in HIV.

[a] Participants receiving this combination either received a penicillin and cephalosporin or two cephalosporins as their standard care for CAP

[b] All participants received their first doses of antibiotics within the first 24-hours of admission. Trial glucocorticoids were initiated within a day of enrolment for all participants

[c] The duration of glucocorticoid administration after participants were discharged was not monitored directly

Table S8: Antibiotic use by trial region

Antibiotic combination	Trial Region		
	Central N= 1057	Coast N=166	Western N=957
Azole	1	1	0
Beta lactam	127	37	81
Beta lactam + Aminoglycoside	2	1	1
Beta lactam + Azole	6	2	5
Beta lactam + Beta lactam[a]	19	11	12
Beta lactam + Lincosamide	1	0	0
Beta lactam + Macrolide	716	96	822
Beta lactam + Nitrofurantoin	1	0	0
Beta lactam + Nitroimidazole	16	9	15
Beta lactam + Sulfonamide	11	3	2
Beta lactam + Tetracycline	132	0	0
Macrolide	10	2	8
Macrolide + Azole	1	0	0
Macrolide + Oxazolidinones	0	0	1
Macrolide + Quinolone	0	0	1
Nitroimidazole	0	0	1
Quinolone + Nitroimidazole	0	1	0
Quinolone + Sulfonamide	1	0	0
Standard care data missing	13	3	8

[a] Participants receiving this combination either received a penicillin and cephalosporin or two cephalosporins as their standard care for CAP

Table S9: Summary of representativeness of trial sites

Category	Detail
Disease under investigation	Community acquired pneumonia
**Considerations relating to generalizability**	
Age	While adults at all ages are susceptible to community acquired pneumonia. A high proportion of those admitted in Kenya are between 25 and 55 years of age (8-12).
Sex	More male adults compared to females are admitted for community acquired pneumonia in Kenya (8-12).
Comorbid conditions	Common comorbid conditions in patients admitted with community acquired pneumonia in Kenya are cardiovascular diseases, diabetes and HIV (11, 12).
Other considerations	Pulmonary tuberculosis is a common co-morbid infection in community acquired pneumonia in Kenya and other countries in sub-Saharan Africa (9, 11, 13).
Overall representativeness of the trial	The participants recruited in this trial represented diverse populations across Kenya. Similar participant characteristics have been reported in large hospital-based surveillance studies in the country (12, 14).<br>The high mortality experienced by trial participants is similar to that reported elsewhere in Kenya (8), Nigeria (15) and Malawi (13).

Table S10: Distribution of chronic comorbidities at enrollment by trial arm

Presence of a comorbidity at enrollment	Control arm	Intervention arm	Total
None	693(63.5%)	699(64.2%)	1392(63.9%)
Present	398(36.5%)	390(35.8%)	788(36.1%)
a. HIV	166(15.2%)	178(16.3%)	344(15.8%)
b. Hypertension	157(14.4%)	143(13.1%)	300(13.8%)
c. Diabetes Mellitus	70(6.4%)	50(4.6%)	120(5.5%)
d. Congestive Cardiac Failure	21(1.9%)	17(1.6%)	38(1.7%)
e. Pulmonary Tuberculosis	10(0.9%)	21(1.9%)	31(1.4%)
f. Cerebral Vascular Accident	7(0.6%)	7(0.6%)	14(0.6%)
g. Malignancy	9(0.8%)	3(0.3%)	12(0.6%)
h. Chronic Kidney Disease	3(0.3%)	3(0.3%)	6(0.3%)
i. Convulsive Disorder	0(0.0%)	5(0.5%)	5(0.2%)
j. Asthma	3(0.3%)	2(0.2%)	5(0.2%)
k. Sickle Cell Disease	2(0.2%)	2(0.2%)	4(0.2%)
l. Arthritis	0(0.0%)	3(0.3%)	3(0.1%)
m. Osteoarthritis	2(0.2%)	1(0.1%)	3(0.1%)
n. Fracture Femur	2(0.2%)	0(0.0%)	2(0.1%)
o. Chronic Leg Ulcer	0(0.0%)	2(0.2%)	2(0.1%)
p. Psychosis	1(0.1%)	1(0.1%)	2(0.1%)
q. *Others	18(1.6%)	13(1.2%)	31(1.4%)

*These participants reported unique chronic comorbidities that couldn’t be grouped together

Table S11: Summary of glucocorticoids received by participants randomized to the intervention arm.

Glucocorticoid mineralocorticoid effect	n (%)	Specific steroid received	n (%)
Low	371 (34.1%)	Dexamethasone - 6mg	371 (34.1%)
Moderate	706 (64.8%)	Methylprednisolone - 30mg	352 (32.3%)
		Prednisolone - 50mg	343 (31.5%)
		*Prednisone - 50mg	11 (1.0%)
High	12 (1.1%)	*Hydrocortisone - 160mg	12 (1.1%)

*The trial stopped randomizing participants to receive hydrocortisone and prednisone after one month of recruitment due to the high pill burden associated with these formulations (see Table S5 above).
15/1089 patients initially received IV glucocorticoids as they were too ill to take oral glucocorticoids. They were distributed as follows: IV dexamethasone-9 patients, IV Hydrocortisone-4 patients, IV Methylprednisolone-1 patient and IV prednisolone-1 patient.

Table S12: Summary of adherence to prescribed medications§

Adherence by study arm	In hospital	Out of hospital		Overall adherence in and out of hospital
		Day 14	Day 30	
Standard Care	97.8	99.9	100.0	99.2
Standard Care plus glucocorticoids	97.7	100.0	99.9	99.2
Dexamethasone - 6mg	97.8	100.0	100.0	99.3
Methylprednisolone - 30mg	97.3	100.0	99.9	99.1
Prednisolone - 50mg	98.0	100.0	100.0	99.3
*Prednisone - 50mg	92.8	100.0	100.0	97.6
*Hydrocortisone - 160mg	98.9	100.0	100.0	99.6
**Adherence by region**				
Coastal	99.1	98.8	98.9	98.9
Central	96.6	100.0	100.0	98.9
Western	98.9	100.0	100.0	99.6

§ Adherence to prescribed medications was monitored directly by the trial clinician during hospitalization. Adherence after discharge from hospital was determined through follow-up phone calls made to trial participants at day 14 and day 30 post-enrolment.
During this phone call, patients or their next of kin were asked the question "Have you been taking all of the medications as prescribed by doctors after leaving hospital?".
Percentages describe the proportion of prescribed medications that were reported to have been taken by the patients and applies to both glucocorticoids and other prescribed medications.
*The trial stopped randomizing participants to receive Hydrocortisone and Prednisone after one month of recruitment due to the high pill burden associated with these formulations (see Table S5 above).

Table S13: Cox regression analysis of mortality at 7-, 14- and 21- days post-enrolment

	Mortality n (%)	Hazard ratio	95% confidence interval
**7 days post enrolment**			
Standard Care	179 (16.4%)	-	-
Standard care plus glucocorticoids	156 (14.3%)	0.86	0.73, 1.00
**14 days post enrolment**			
Standard Care	229 (21.0%)	-	-
Standard care plus glucocorticoids	203(18.6%)	0.87	0.74, 1.01
**21 days post enrolment**			
Standard Care	259 (23.7%)	-	-
Standard care plus glucocorticoids	224 (20.6%)	0.84	0.73, 0.98

Table S14: In-hospital and out-of-hospital mortality by trial arm

Trial arm	In hospital	Out of hospital
Standard Care	223/1091(20.4%)	61/1091(5.6%)
Standard Care plus glucocorticoids	196/1089(18.0%)	50/1089(4.6%)

Table S15: Results of complete case analysis and modified intention to treat (ITT) analysis of the primary outcome

	Mortality n/N (%)	Hazard ratio	95% confidence interval
[1] **Complete case analysis (N= 2082)**			
Standard Care	284/1036 (27.4%)	-	-
Standard care plus glucocorticoids	246/1046 (23.5%)	0.84	0.73, 0.96
[2] **Modified ITT population (N= 2142)**			
Standard Care	272/1072 (25.4%)	-	-
Standard care plus glucocorticoids	233/1070 (21.8%)	0.83	0.72, 0.97

[1] Complete case analysis (specified posthoc) excludes participants whose 30-day outcome data was missing (N=98) due to loss to follow-up (N=23) or withdrawal from the trial (N=75). See Table S1 for characteristics of these participants. In the primary intention to treat analysis the data were censored at the time of the last observation.

[2] Modified ITT population (specified posthoc) excluded 38 participants who were found to have received glucocorticoids prior to randomization

Table S16: Number of deaths per trial site

Trial site	Mortality<br>Control arm (n=284)	Mortality<br>Intervention arm (n=246)
1. Kilifi County Hospital	7/13 (53.8)	0/7 (0.0)
2. Coast General Teaching and Referral Hospital	23/72 (31.9)	23/74 (31.1%)
3. Machakos County Referral Hospital	30/81 (37.0%)	20/76 (26.3%)
4. Kenyatta National Hospital	-	-
5. Mbagathi sub-County Hospital	4/30 (13.3%)	5/26 (19.2%)
6. Mama Lucy Kibaki Hospital	6/46 (13.0%)	4/39 (10.3%)
7. Kenyatta University Teaching Research and Referral Hospital	1/21 (4.8%)	5/21 (23.8%)
8. Kiambu County Referral Hospital	11/49 (22.4%)	9/71 (12.7%)
9. Naivasha County Hospital	51/240 (21.2%)	33/231 (14.3%)
10. Nakuru County Referral Hospital	14/61 (23.0%)	15/65 (23.1%)
11. Kisii County Referral Hospital	7/31 (22.6%)	8/36 (22.2%)
12. Kisumu County Hospital	12/71 (16.9%)	13/69 (18.8%)
13. Jaramogi Oginga Odinga Teaching and Referral Hospital	3/23 (13.0%)	6/20 (30.0%)
14. Kakamega County Teaching and Referral Hospital	21/63 (33.3)	20/55 (36.4%)
15. Moi Teaching and Referral Hospital	-	-
16. Kitale County Hospital	9/42 (21.4%)	13/43 (30.2%)
17. Bungoma County Hospital	77/217 (35.5%)	71/231 (30.7%)
18. Busia County Hospital	8/31 (25.8%)	1/25 (4.0%)

Table S17: Summary of all adverse events reported in the trial.

Diagnosis	Control arm n=174 (%)	Intervention arm n=211 (%)	Total n=385 (%)
Pulmonary Tuberculosis	35 (20.1%)	34 (16.1%)	69 (17.9%)
Hyperglycemia	3 (1.7%)	35 (16.6%)	38 (9.9%)
Acute Kidney injury	14 (8.0%)	15 (7.1%)	29 (7.5%)
Anemia	13 (7.5%)	11 (5.2%)	24 (6.2%)
Human Immunodeficiency Virus	8 (4.6%)	7 (3.3%)	15 (3.9%)
Heart Failure	5 (2.9%)	10 (4.7%)	15 (3.9%)
Respiratory Distress	6 (3.4%)	5 (2.4%)	11 (2.9%)
Hypertension	5 (2.9%)	5 (2.9%)	10 (2.6%)
Chronic Kidney Disease	6 (3.4%)	2 (0.9%)	8 (2.1%)
Severe Community Acquired Pneumonia	4 (2.3%)	4 (1.9%)	8 (2.1%)
Pleural Effusion	5 (2.9%)	2 (0.9%)	7 (1.8%)
Candidiasis	3 (1.7%)	3 (1.4%)	6 (1.6%)
Gastritis	4 (2.3%)	2 (0.9%)	6 (1.6%)
Malignancy	4 (2.3%)	2 (0.9%)	6 (1.6%)
Vomiting	2 (1.1%)	4 (1.9%)	6 (1.6%)
Cryptococcal Meningitis	3 (1.7%)	2 (0.9%)	5 (1.3%)
Headache	3 (1.7%)	2 (0.9%)	5 (1.3%)
Hypotension	2 (1.1%)	3 (1.4%)	5 (1.3%)
Pneumocystis Carinii Pneumonia	2 (0.9%)	3 (1.4%)	5 (1.3%)
Extrapulmonary tuberculosis	4 (1.1%)	0 (0.0%)	4 (1.0%)
Malaria	2 (1.1%)	2 (0.9%)	4 (1.0%)
Peptic Ulcer Disease	2 (1.1%)	2 (0.9%)	4 (1.0%)
Pulmonary Embolism	2 (0.9%)	2 (0.9%)	4 (1.0%)
Cardiomyopathy	0 (0.0%)	3 (1.4%)	3 (0.8%)
Chronic obstructive pulmonary disease	1 (0.6%)	2 (0.9%)	3 (0.8%)
Constipation	2 (1.1%)	1 (0.5%)	3 (0.8%)
Convulsive disorder	1 (0.6%)	2 (0.9%)	3 (0.8%)

Pulmonary Hypertension	2 (0.9%)	1 (0.5%)	3 (0.8%)
Tachycardia	2 (1.1%)	1 (0.5%)	3 (0.8%)
Venous Thromboembolism	2 (1.1%)	1 (0.5%)	3 (0.8%)
Alcohol withdrawal	0 (0.0%)	2 (0.9%)	2 (0.5%)
Atrial Fibrillation	0 (0.0%)	2 (0.9%)	2 (0.5%)
Cellulitis	2 (1.1%)	0 (0.0%)	2 (0.5%)
Cerebrovascular accident	2 (1.1%)	0 (0.0%)	2 (0.5%)
Chest pain	0 (0.0%)	2 (0.9%)	2 (0.5%)
Gastroenteritis	1 (0.6%)	1 (0.5%)	2 (0.5%)
Hepatitis	0 (0.0%)	2 (0.9%)	2 (0.5%)
Hyperkalemia	1 (0.6%)	1 (0.5%)	2 (0.5%)
Hyperthyroidism	0 (0.0%)	2 (0.9%)	2 (0.5%)
Hypoglycemia	1 (0.6%)	1 (0.5%)	2 (0.5%)
Hypokalemia	1 (0.6%)	1 (0.5%)	2 (0.5%)
Low back pain	1 (0.6%)	1 (0.5%)	2 (0.5%)
Meningitis	0 (0.0%)	2 (0.9%)	2 (0.5%)
Neuropathy	0 (0.0%)	2 (0.9%)	2 (0.5%)
Acute Liver Disease	1 (0.6%)	0 (0.0%)	1 (0.3%)
Acute pancreatitis	1 (0.6%)	0 (0.0%)	1 (0.3%)
Acute psychosis	1 (0.6%)	0 (0.0%)	1 (0.3%)
Alcoholic hepatitis	0 (0.0%)	1 (0.5%)	1 (0.3%)
Altered consciousness	0 (0.0%)	1 (0.5%)	1 (0.3%)
Arthritis	0 (0.0%)	1 (0.5%)	1 (0.3%)
Brain atrophy	0 (0.0%)	1 (0.5%)	1 (0.3%)
Bronchiectasis	0 (0.0%)	1 (0.5%)	1 (0.3%)
Cholecystitis	0 (0.0%)	1 (0.5%)	1 (0.3%)
Common Cold	1 (0.6%)	0 (0.0%)	1 (0.3%)
COVID-19	0 (0.0%)	1 (0.5%)	1 (0.3%)
Deep Venous Thrombosis	0 (0.0%)	1 (0.5%)	1 (0.3%)
Delirium	1 (0.6%)	0 (0.0%)	1 (0.3%)

Dermatitis	1 (0.6%)	0 (0.0%)	1 (0.3%)
Diabetes Mellitus	0 (0.0%)	1 (0.5%)	1 (0.3%)
Diarrhea	1 (0.6%)	0 (0.0%)	1 (0.3%)
Diffuse Interstitial Lung Disease	0 (0.0%)	1 (0.5%)	1 (0.3%)
Empyema	0 (0.0%)	1 (0.5%)	1 (0.3%)
Enteritis	1 (0.6%)	0 (0.0%)	1 (0.3%)
Epistaxis	0 (0.0%)	1 (0.5%)	1 (0.3%)
Erectile dysfunction	0 (0.0%)	1 (0.5%)	1 (0.3%)
Esophageal Varices	1 (0.6%)	0 (0.0%)	1 (0.3%)
Fever	0 (0.0%)	1 (0.5%)	1 (0.3%)
Generalized Edema	1 (0.6%)	0 (0.0%)	1 (0.3%)
Gout	1 (0.6%)	0 (0.0%)	1 (0.3%)
Hearing loss	0 (0.0%)	1 (0.5%)	1 (0.3%)
Hemothorax	1 (0.6%)	0 (0.0%)	1 (0.3%)
Hypothermia	1 (0.6%)	0 (0.0%)	1 (0.3%)
Jaundice	1 (0.6%)	0 (0.0%)	1 (0.3%)
Leg Pain	0 (0.0%)	1 (0.5%)	1 (0.3%)
Loss of Appetite	0 (0.0%)	1 (0.5%)	1 (0.3%)
Loss of consciousness	0 (0.0%)	1 (0.5%)	1 (0.3%)
Myocardial infarction	0 (0.0%)	1 (0.5%)	1 (0.3%)
Nausea	0 (0.0%)	1 (0.5%)	1 (0.3%)
Olecranon Bursitis	1 (0.6%)	0 (0.0%)	1 (0.3%)
Pneumothorax	1 (0.6%)	0 (0.0%)	1 (0.3%)
Renal Cyst	0 (0.0%)	1 (0.5%)	1 (0.3%)
Renal Failure	1 (0.6%)	0 (0.0%)	1 (0.3%)
Sepsis	1 (0.6%)	0 (0.0%)	1 (0.3%)
Sickle Cell Disease	0 (0.0%)	1 (0.5%)	1 (0.3%)
Toxoplasmosis	0 (0.0%)	1 (0.5%)	1 (0.3%)
Urinary Tract Infection	0 (0.0%)	1 (0.5%)	1 (0.3%)

Adverse events have been listed in descending order of frequency

Table S18: Summary of all serious adverse events reported in the trial

Diagnosis	Control arm n (%)	Intervention arm n (%)	Total n (%)
Severe Community Acquired Pneumonia	5 (6.6%)	8 (10.5%)	13 (17.1%)
Pulmonary Embolism	7 (9.2%)	3 (4.0%)	10 (13.2%)
Pulmonary Tuberculosis	6 (7.9%)	3 (4.0%)	9 (11.9%)
Chronic Kidney Disease	6 (7.9%)	1 (1.3%)	7 (9.2%)
Lung Abscess	7 (9.2%)	0 (0.0%)	7 (9.2%)
Malignancy	5 (6.6%)	1 (1.3%)	6 (7.9%)
Meningitis / Meningoencephalitis	2 (2.6%)	2 (2.6%)	4 (5.3%)
Cerebrovascular Accident	1 (1.3%)	2 (2.6%)	3 (3.9%)
Pleural Effusion	2 (2.6%)	1 (1.3%)	3 (3.9%)
Hyperglycemia	0 (0.0%)	3 (4.0%)	3 (4.0%)
Peptic Ulcer Disease	2 (2.6%)	0 (0.0%)	2 (2.6%)
Acute Kidney Injury	0 (0.0%)	2 (2.6%)	2 (2.6%)
Sepsis	0 (0.0%)	2 (2.6%)	2 (2.6%)
Other Cardiovascular Disease	1 (1.3%)	1 (1.3%)	2 (2.6%)
Heart Failure	0 (0.0%)	1 (1.3%)	1 (1.3%)
Brain Edema	0 (0.0%)	1 (1.3%)	1 (1.3%)
Fatigue	1 (1.3%)	0 (0.0%)	1 (1.3%)
Pulmonary Hypertension	0 (0.0%)	1 (1.3%)	1 (1.3%)
Renal Failure	0 (0.0%)	1 (1.3%)	1 (1.3%)
Intestinal Obstruction	0 (0.0%)	1 (1.3%)	1 (1.3%)
Appendicitis	1 (1.3%)	0 (0.0%)	1 (1.3%)
Anemia	0 (0.0%)	1 (1.3%)	1 (1.3%)
Fever	0 (0.0%)	1 (1.3%)	1 (1.3%)
Hypertension	1 (1.3%)	0 (0.0%)	1 (1.3%)
Immune Thrombocytopenic Purpura	0 (0.0%)	1 (1.3%)	1 (1.3%)
Malaria	0 (0.0%)	1 (1.3%)	1 (1.3%)

Serious adverse events have been listed in descending order of frequency

Interim Analysis Report

A pragmatic randomized controlled trial of standard care versus steroids plus standard care for treatment of pneumonia in adults admitted to Kenyan hospitals (SONIA)

Interim Analysis Report

Protocol Number:	SERU 4319
Protocol Version:	Version 2.0 1st February 2022
Trial registration number	PACTR202111481740832
Principal Investigator:	Dr Anthony Etyang
Investigational product	Systemic steroids: Betamethasone, Dexamethasone, Hydrocortisone, Methyl prednisone, Prednisolone, Prednisone
Funder:	Wellcome Trust (UK)<br>Gibbs Building<br>215 Euston Road<br>London NW1 2BE UK
Statisticians	Benedict Orindi, Paul Mwaniki
Date report issued:	12/04/2023
Data cutoff date:	21/01/2023

Introduction

It is known that if adjunctive steroid therapy is beneficial in community-acquired pneumonia. Studies conducted elsewhere in the world have yielded conflicting results, and in those that suggested a benefit, this was limited to patients with severe disease. Dexamethasone was shown to be beneficial in patients with confirmed Coronavirus Disease (COVID-19) who had severe disease. The study showing benefit had most patients recruited in Europe. The World Health Organization (WHO) subsequently revised treatment guidelines to recommend the use of steroids in patients with severe COVID-19. COVID-19 testing remains low in low and middle-income countries such as Kenya; patients with acute respiratory signs and symptoms, possibly due to COVID-19 infection, do not always get tested. In the absence of routine testing for COVID-19, it is likely that clinicians will consider prescribing adjunctive steroids to patients with community-acquired pneumonia because of their proven benefit in patients with COVID-19. The SONIA trial is a pragmatic open-label randomized trial that seeks to determine whether the addition of low-dose steroids to standard treatment in patients with CAP is associated with decreased mortality.

The primary objective is to determine if there is a difference in the proportion of patients with community acquired pneumonia that die within 30 days of being randomized to receive adjunctive steroid treatment compared to those that are randomized to receive standard treatment only.

The secondary objectives are:

To determine if there is a difference in the proportion of patients with community acquired pneumonia that die within 7, 14 and 21 days respectively of being randomized to receive adjunctive steroid treatment compared to those that are randomized to receive standard treatment only;

To determine if there is a difference in the proportion of patients with community acquired pneumonia that die in hospital and out of hospital, respectively, after being randomized to receive adjunctive steroid treatment compared to those that are randomized to receive standard treatment only;

To determine if there is a difference in the time to death of patients randomized to receive adjunctive steroid treatment compared to those on standard care alone;

Profile the immune and metabolic status before treatment and induced changes 24, 48 and 72 hours after treatment, to determine the correlation of pre-existing and treatment induced changes with study outcomes.

The primary hypothesis is that adjunct corticosteroid treatment will reduce mortality in the intervention arm by ~25% (relative measure).

This report presents the interim results for the trial. We summarize enrollment, demographic and baseline characteristics, and primary endpoint data.

Trial treatment

Patients randomized to standard of care received treatment according to local guidelines for community acquired pneumonia (CAP) which currently recommend a beta lactam antibiotic and a macrolide for 7–14 days [1,2]. Patients who were randomized to the intervention arm received the standard of care treatment for CAP as outlined above AND any of the following equivalent doses of steroids for a period of 10 days [3]:

Steroid	Once daily dose
Dexamethasone	6mg
Betamethasone	5mg
Hydrocortisone	160mg
Methylprednisolone	30mg
Prednisolone	50mg
Prednisone	50mg

Assessment of interim results

As indicated in the study protocol, statistical analysis plan (SAP) and the Data and Safety Monitoring Board (DSMB) charter, the DSMB will review the data presented here. The analysis explores the primary endpoint, which is to determine the effect of adjunctive steroids on all-cause mortality at 30 days after randomization. Based on the results, the DSMB will share a recommendation on the continuation of the trial based on established rules for conducting interim analyses with the Trial Steering Committee (TSC). The DSMB will consider the 'totality of evidence' before their recommendation is made. This will include a review of the nature and implications of the results, implication of collection of additional data (budget, risk to future participants) and the importance of further sub-group analyses.

Analysis plan and key points

The following points were adopted from the Statistical Analysis Plan version 3.0 dated 2nd January 2023.

Sample Size Calculation

A conservative assumption that 30-day mortality in the control arm of the trial will be 20% and that 30-day mortality in the intervention arm will be 15% was used to calculate the study's sample size. Allowing for 5% loss to follow-up, a sample size of 1 090 patients per study arm (total 2 180 patients, ~ 390 deaths) will achieve statistical power of 85%, with a 2-sided type 1 error rate of 5%.

Interim Analysis Methods

We first estimated the proportion that died 30 days after randomization by treatment arm. These were presented together with their 95% Agresti-Coull confidence intervals (CIs). A Cox regression model was used to estimate the hazard ratio (HR) comparing 30-day mortality between the randomized groups and its confidence interval. Those not experiencing the event were censored at 30 days post-randomization.

Kaplan-Meier survival curves were constructed to display cumulative mortality over the 30-day follow-up period and the curves compared using the Wilcoxon-Breslow-Gehan test. Proportional hazards assumption was evaluated using graphical (i.e., Schoenfeld residuals, log-log plots and observed versus predicted plots) and goodness-of-fit (GOF) approaches [4]. All analyses were performed using Stata version 15.1 (StataCorp, College Station, TX).

Demographic and baseline characteristics

Between 21st April 2022 and 30th January 2023, we screened and enrolled 844 participants (Group 1 = 443, Group 2 = 401). Of these, 80% had completed their final study visit by the date. About 5% of all recruited participants have been withdrawn from the trial.

The withdrawals were mainly due to a change in the eligibility criteria; participants found to be COVID-19 positive or those in whom the steroids were stopped because of other conditions e.g. newly diagnosed COPD or asthma. 1 participant withdrew consent because the participant did not want further blood sampling. Breakdown of recruitment summary is presented in Table 1.

Table 1. Subject enrolment status by treatment arm. Data are number (%).

Trial Activity	Trial Arm, n (%)		
	Group 1	Group 2	Combined
Participants consented	517 (52%)	478 (48%)	995 (100%)
Study visits (% enrolled)			
Completed day 30	443 (86%)	401 (84%)	844 (85%)
LTFU/Withdrawals			
Lost to follow-up	2 (<1%)	1 (<1%)	3 (<1%)
Withdrawals (% enrolled)	22 (4%)	18 (4%)	40 (4%)
Summary			
Completed the study/withdrawn/ltfu	467 (90%)	420 (88%)	887 (89%)
In active follow up (% enrolled)	50 (10%)	58 (12%)	108 (11%)

The 844 participants comprised males and females aged 18–108 years. In general, the participants were balanced between the two trial arms with respect to the demographics and baseline characteristics (Table 2).

Table 2. Demographics and baseline characteristics by study arm

	Group 1	Group 2	Combined
Number enrolled	443 (52.5%)	401 (47.5%)	844 (100%)
Age, years, mean (SD)	54.8 (20.7)	54.7 (21.3)	54.8 (21.0)
Sex			
Male, n (%)	203 (45.8%)	183 (45.6%)	386 (45.7%)
Female, n (%)	240 (54.2%)	218 (54.4%)	458 (54.3%)

Mortality at 30 days after randomization

Of the 844 participants, the proportion that died by day 30 after randomization was 28% (95% CI: 24–33%) in the group 1 compared to 26% (95% CI: 22-31%) in group 2, yielding a risk ratio of 0.89 (95% CI: 0.68-1.17) (Table 3). Figure 1 presents the estimated curves by treatment arm over the 30-day period. It shows late non-significant lower mortality in group 2 compared to those in group 1

(Wilcoxon-Gehan test p=0.559). Results indicated that the PH assumption was not violated using the GOF test (p= 0.573) and graphical assessments.

Table 3: Mortality at 30 days post-randomization by study arm.

Study arm	N	Number (%) dead	Mortality rate	Crude rate ratio (95% CI)	p value
Group 1	443	125 (28.2%)	0.012		
Group 2	401	105 (26.2%)	0.010	0.89 (0.68-1.17)	0.413

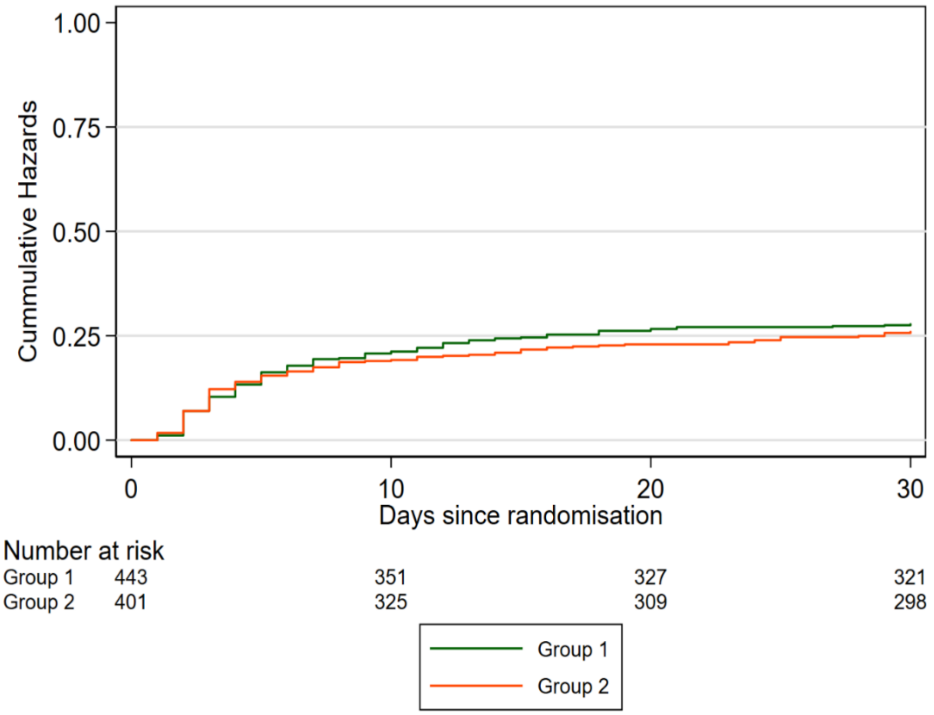

Figure 1: Cumulative hazard for participants in group 1 versus those in group 2 within 30 days of randomization.

Conclusions

While patients in group 2 seem to have a lower rate of death compared to those group 1 within 30 days of randomization, a 15% or higher difference in mortality across the two groups is required for the difference to be considered clinically significant.

References

1. Achieng L, Menge T, Omonge E. The KNH Guide to Empiric Antimicrobial Therapy: Kenyatta National Hospital and University of Nairobi; 2018.
2. Clinical Guidelines for Management and Referral of Common Conditions at Levels 4-6: Hospitals. Ministry of Health, 2009. (Accessed 08 July 2013, at http://www.pharmacyboardkenya.org/assets/files/Clinical%20Guidelines%20Vol%20III%20Final.pdf.)
3. Liu D, Ahmet A, Ward L, et al. A practical guide to the monitoring and management of the complications of systemic corticosteroid therapy. Allergy Asthma Clin Immunol 2013;9:30.
4. Kleinbaum, D. G., and Klein, M. (1996). *Survival analysis a self-learning text*. Springer

Author contributions

RKL, HG, BO, MS, AIA, EWK, ME, MH, LIO-O, DK, PB, AA, SA and AOE were involved in the design of the study. RKL, HG, PM, BO, EOO, SM, LM, LI, JS, MS, EI, INO, AW, EM, HK, PA, JN, CW, AO, HB, SBO, WO, DMM, WL, BM, IA, CM, MS, NAA, BG, MGM, CAO, LAO, NK, LO, AA, SA and AOE were involved in the conduct of the study. RKL, HG, PM, BO, EOO and AOE were involved in analysis and preparation of the draft manuscript.

RKL and AOE wrote the first draft of the manuscript. All authors contributed to data interpretation and final approval of the manuscript.

References

1. Little RJA. A Test of Missing Completely at Random for Multivariate Data with Missing Values. Journal of the American Statistical Association. 1988;83(404):1198-202.
2. Little RJ, Rubin DB. Statistical analysis with missing data: John Wiley & Sons; 2019.
3. Schulz KF, Grimes DA. Sample size slippages in randomised trials: exclusions and the lost and wayward. The Lancet. 2002;359(9308):781-5.
4. Fine Michael J, Auble Thomas E, Yealy Donald M, Hanusa Barbara H, Weissfeld Lisa A, Singer Daniel E, et al. A Prediction Rule to Identify Low-Risk Patients with Community-Acquired Pneumonia. New England Journal of Medicine.336(4):243-50.
5. Barasa EW, Ouma PO, Okiro EA. Assessing the hospital surge capacity of the Kenyan health system in the face of the COVID-19 pandemic. PloS one. 2020;15(7):e0236308-e.
6. Williams DM. Clinical Pharmacology of Corticosteroids. Respiratory Care. 2018;63(6):655-70.
7. Liu D, Ahmet A, Ward L, Krishnamoorthy P, Mandelcorn ED, Leigh R, et al. A practical guide to the monitoring and management of the complications of systemic corticosteroid therapy. Allergy, Asthma & Clinical Immunology. 2013;9(1):30.
8. Etyang AO, Munge K, Bunyasi EW, Matata L, Ndila C, Kapesa S, et al. Burden of disease in adults admitted to hospital in a rural region of coastal Kenya: an analysis of data from linked clinical and demographic surveillance systems. The lancet global health. 2014;2(4):e216-e24.
9. Scott JAG, Hall AJ, Muyodi C, Lowe B, Ross M, Chohan B, et al. Aetiology, outcome, and risk factors for mortality among adults with acute pneumonia in Kenya. The Lancet. 2000;355(9211):1225-30.
10. Muthumbi E, Lowe BS, Muyodi C, Getambu E, Gleeson F, Scott JAG. Risk factors for community-acquired pneumonia among adults in Kenya: a case-control study. Pneumonia (Nathan). 2017;9:17.
11. Nambafu J, Achakolong M, Mwendwa F, Bwika J, Riunga F, Gitau S, et al. A prospective observational study of community acquired pneumonia in Kenya: the role of viral pathogens. BMC Infect Dis. 2021;21(1):703.
12. Lucinde RK, Gathuri H, Isaaka L, Ogero M, Mumelo L, Kimego D, et al. Prospective clinical surveillance for severe acute respiratory illness and COVID-19 vaccine effectiveness in Kenyan hospitals during the COVID-19 pandemic. BMC Infect Dis. 2024;24(1):1246.
13. Aston SJ, Ho A, Jary H, Huwa J, Mitchell T, Ibitoye S, et al. Etiology and risk factors for mortality in an adult community-acquired pneumonia cohort in Malawi. American journal of respiratory and critical care medicine. 2019;200(3):359-69.
14. Ogero M, Isaaka L, Mumelo L, Kimego D, Njoroge T, Mbevi G, et al. Effects of the COVID-19 pandemic on hospital admissions and inpatient mortality in Kenya. medRxiv. 2022.

15. Iroezindu MO, Isiguzo GC, Chima EI, Mbata GC, Onyedibe KI, Onyedum CC, et al. Predictors of in-hospital mortality and length of stay in community-acquired pneumonia: a 5-year multi-centre case control study of adults in a developing country. Transactions of The Royal Society of Tropical Medicine and Hygiene. 2016;110(8):445-55.
16. Ayieko P, Ogero M, Makone B, Julius T, Mbevi G, Nyachiro W, et al. Characteristics of admissions and variations in the use of basic investigations, treatments and outcomes in Kenyan hospitals within a new Clinical Information Network. Arch Dis Child. 2016;101(3):223.
